# Apigenin as an emerging hepatoprotective agent: current status and future perspectives

**DOI:** 10.3389/fphar.2024.1508060

**Published:** 2024-12-19

**Authors:** Cheng Wang, Xiaoli Feng, Wen Li, Li Chen, Xinming Wang, Yimiao Lan, Rong Tang, Ting Jiang, Lingli Zheng, Gang Liu

**Affiliations:** ^1^ School of Clinical Medical, Chengdu Medical College, Chengdu, China; ^2^ Department of Pharmacy, The First Affiliated Hospital of Chengdu Medical College, Chengdu, China; ^3^ Department of Respiratory and Critical Care Medicine, The First Affiliated Hospital of Chengdu Medical College, Chengdu, China; ^4^ College of Foreign Languages and Cultures, Sichuan University, Chengdu, China

**Keywords:** apigenin, liver disease, hepatoprotection, toxicity, pharmacokinetics, new formulations

## Abstract

Apigenin (C_15_H_10_O_5_, API) is a natural flavonoid widely found in vegetables, fruits, and plants such as celery, oranges, and chamomile. In recent years, API has attracted considerable attention as a dietary supplement due to its low toxicity, non-mutagenic properties and remarkable therapeutic efficacy in various diseases. In particular, evidence from a large number of preclinical studies suggests that API has promising effects in the prevention and treatment of a variety of liver diseases, including multifactorial liver injury, non-alcoholic fatty liver disease/non-alcoholic steatohepatitis, liver fibrosis and liver cancer. This paper provides a comprehensive review of the progress of research into the therapeutic applications of API in liver diseases as of August 2024, based on literature retrieved from databases such as Web of Science, PubMed, CNKI, Google Scholar and ScienceDirect. The hepatoprotective effects of API involve multiple molecular mechanisms, including inhibition of inflammation, alleviation of hepatic oxidative stress, amelioration of insulin resistance, promotion of fatty acid oxidation, inhibition of liver cancer cell proliferation and differentiation, and induction of tumour cell apoptosis. More importantly, signaling pathways such as Nrf2, NF-κB, PI3K/Akt/mTOR, NLRP3, Wnt/β-catenin, TGF-β1/Smad3, AMPK/SREBP, PPARα/γ, MAPKs, and Caspases are identified as key targets through which API exerts its beneficial effects in various liver diseases. Studies on its toxicity and pharmacokinetics indicate that API has low toxicity, is slowly metabolized and excreted *in vivo*, and has low oral bioavailability. In addition, the paper summarises and discusses the sources, physicochemical properties, new dosage forms, and current challenges and opportunities of API, with the aim of providing direction and rationale for the further development and clinical application of API in the food, pharmaceutical and nutraceutical fields.

## Graphical Abstract

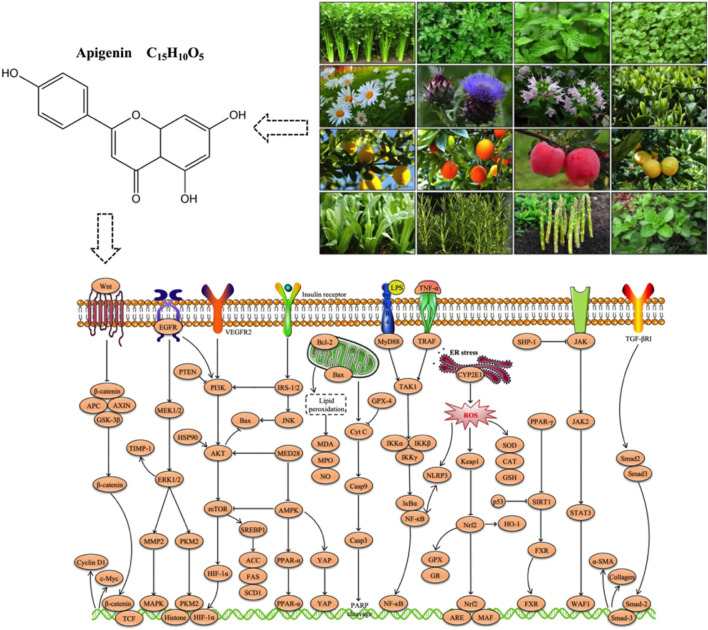

## 1 Introduction

The liver is a crucial metabolic organ essential for maintaining normal physiological functions, including detoxification, protein synthesis, lipid metabolism, bile secretion, and glucose regulation (Kubes and Jenne, 2018; Zaefarian et al., 2019). Dysfunction of the liver not only disrupts the body’s metabolic balance but can also lead to systemic diseases (Ginès et al., 2022). Unfortunately, liver diseases have become a significant global health challenge, with pathologies ranging from non-alcoholic fatty liver disease (NAFLD) and alcoholic liver disease (ALD) to viral hepatitis, liver fibrosis, and hepatocellular carcinoma (HCC) (Ginès et al., 2022; Devarbhavi et al., 2023). Despite remarkable advances in modern medicine in the diagnosis and treatment of liver diseases, current therapeutic approaches still face limitations, including suboptimal efficacy, significant drug side effects and considerable inter-individual variability (Neshat et al., 2021). In chronic liver diseases, the interplay between inflammation, oxidative stress, and apoptosis often exacerbates the condition, leading to progressive deterioration (Szabo and Petrasek, 2015; Wang et al., 2021b; Allameh et al., 2023). Therefore, the development of safe, effective, and multi-targeted therapeutic strategies is of paramount importance.

Natural products, known for their diverse biological activities and low toxicity, have become a research focus in recent years (Wang et al., 2021a; Dai et al., 2023; Wang et al., 2023). Apigenin (API), a naturally occurring flavonoid, has attracted significant attention due to its broad pharmacological activities, particularly its potential in liver protection (Salehi et al., 2019). Widely distributed in various vegetables, medicinal plants, and fruits, API exhibits notable antioxidant, anti-inflammatory, anti-fibrotic, and anti-tumor activities, positioning it as a promising candidate for the treatment of liver diseases (Alshehri et al., 2019). Research has shown that API exerts its hepatoprotective effects through multiple mechanisms, including inhibition of inflammatory responses, alleviation of hepatic oxidative stress, improvement of insulin resistance, regulation of lipid metabolism, inhibition of liver cancer cell proliferation and differentiation, and induction of tumor cell apoptosis (Chiang et al., 2006; Wang et al., 2017; Goudarzi et al., 2021; Chou et al., 2024; Hsu et al., 2024). More importantly, studies have identified key signaling pathways involved in API-mediated liver protection, including nuclear factor erythroid 2-related factor 2 (Nrf2), nuclear factor kappa-B (NF-κB), phosphatidylinositide 3-kinase (PI3K)/protein kinase B (AKT)/mammalian target of rapamycin (mTOR), NOD-like receptor family pyrin domain containing 3 (NLRP3), Wnt/β-catenin, farnesoid X receptor (FXR), transforming growth factor-β1 (TGF-β1)/mothers against decapentaplegic homolog 3 (Smad3), and peroxisome proliferator-activated receptor alpha (PPARα) (Feng et al., 2017; Yang J. et al., 2018; Yue et al., 2020; Ji et al., 2021; Pan et al., 2021; Zheng et al., 2021; Meng et al., 2022).

However, there is currently a lack of systematic reviews on the sources, physicochemical properties, hepatoprotective effects, toxicity, pharmacokinetics, and novel formulations of API. Therefore, this paper aims to provide a comprehensive review of the research progress on API in the treatment of liver diseases up to August 2024, by searching databases such as Web of Science, PubMed, CNKI, Google Scholar, and ScienceDirect. In addition, this review will reflect on the limitations of existing research and propose future research directions to provide a solid theoretical foundation for the clinical application of API.

## 2 Sources and characteristics of API

**FIGURE 1:**
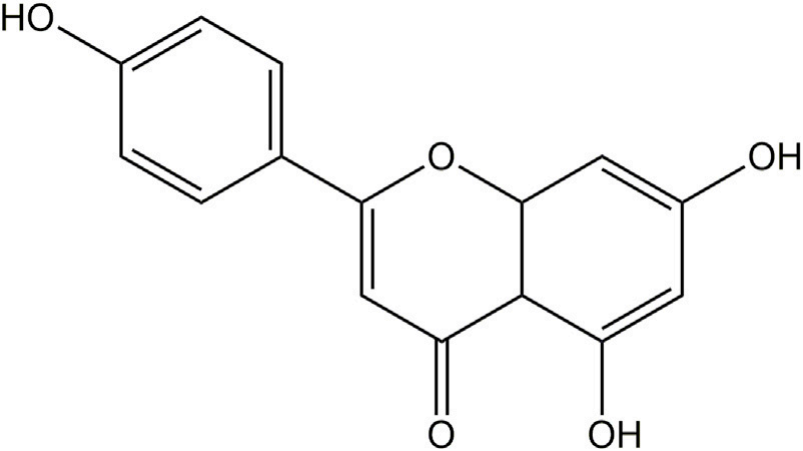
Chemical structure of API.

**FIGURE 2:**
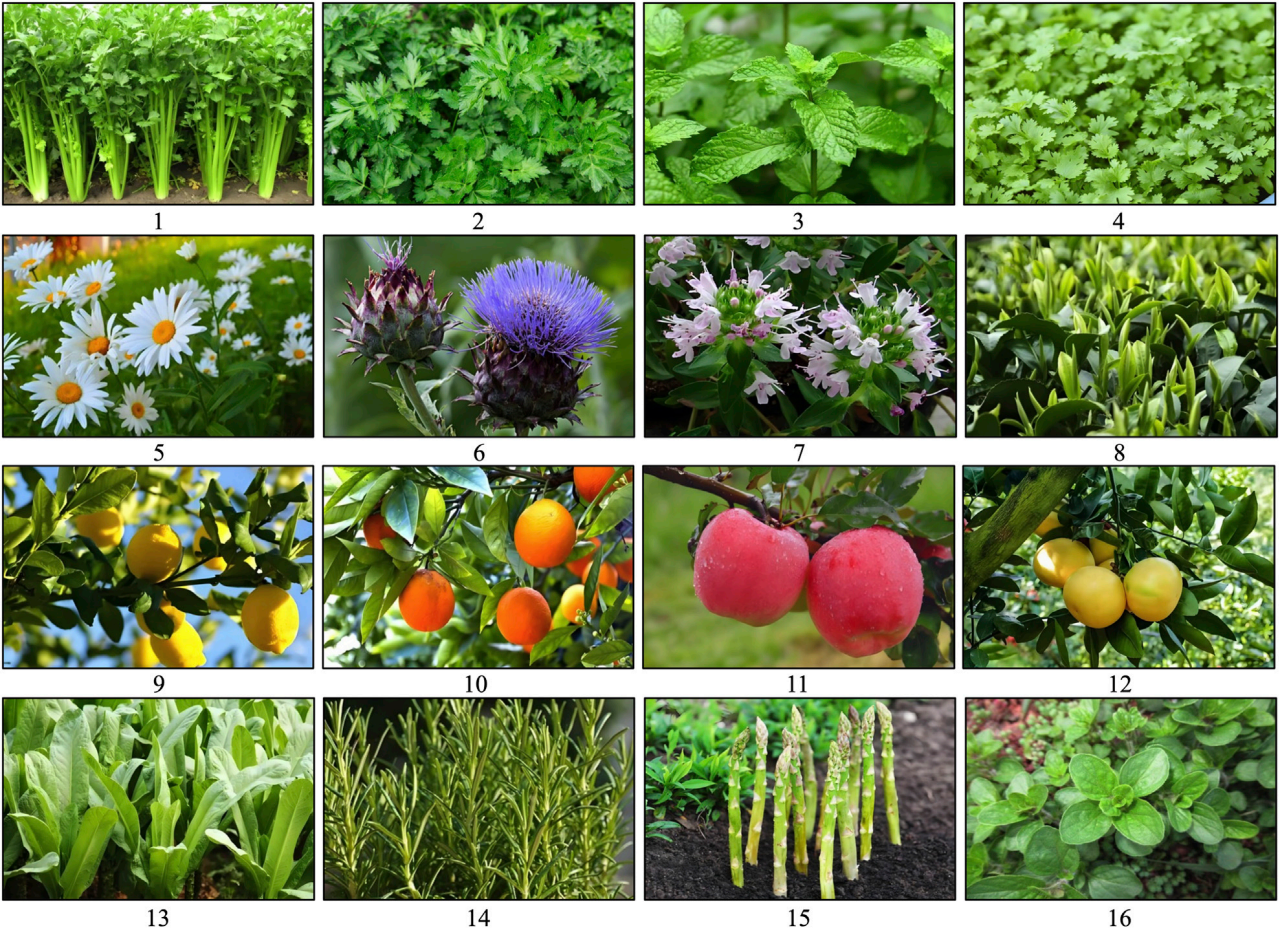
Sources of API. 1: Celery; 2: Parsley; 3. Mint; 4: Coriander; 5: Chamomile; 6: Artichoke; 7: Thyme; 8: Green tea; 9: Lemon; 10: Orange; 11. Apple; 12: Grapefruit; 13: Lettuce; 14: Rosemary; 15: Asparagus; 16: Oregano. Referring to the picture is from the website: http://www.gbif.org; https://image.baidu.com.

API (C_15_H_10_O_5_, Figure 1) is a naturally occurring flavonoid, often referred to as a “phytoestrogen.” It is widely found in various plants, vegetables, and fruits, including parsley, celery, mint, chamomile, thyme, lettuce, asparagus, oranges, and grapefruit, with particularly high concentrations in parsley and celery (Figure 2) (Madunić et al., 2018; Alshehri et al., 2019). Studies have shown that API extracted from celery constitutes 17% of the total flavonoid content (Crozier et al., 1997). In recent years, API has garnered increasing attention as a dietary supplement due to its low toxicity, non-mutagenic properties, and significant therapeutic efficacy in various diseases.

Physically, API is a yellow crystalline powder with a molecular weight of 270.24 g/mol and a density of 1.548 g/cm³. API has very low solubility in water. In contrast, it has good solubility in organic solvents such as ethanol, potassium hydroxide, and dimethyl sulfoxide (Tang et al., 2017). These physical properties limit its application in aqueous systems. However, its biological activity can be fully utilized through proper solvent selection. Chemically, API contains three phenolic hydroxyl groups and two carbon-carbon double bonds, which impart strong antioxidant activity (Zhou et al., 2023). API effectively scavenges free radicals, inhibits lipid peroxidation, and demonstrates significant anti-inflammatory, anticancer, and antimicrobial potential (Wang et al., 2014; Yang J. et al., 2018; Zarei et al., 2021). Moreover, the phenolic hydroxyl groups of API can chelate metal ions, further enhancing its antioxidant capacity (Spiegel and Sroka, 2023).

In summary, API shows potential for a wide range of applications in the food, pharmaceutical and nutraceutical fields due to its excellent antioxidant and other bioactive activities. Detailed data on the physical and chemical properties of API are presented in Table 1. Data obtained from https://china.guidechem.com.

**TABLE 1:** Physical and chemical properties of API.

Name	Apigenin
Alias	5,7-Dihydroxy-2-(4-hydroxyphenyl)-4H-1-benzopyran-4-one
Source	Various fruits, vegetables and herbs
CAS number	520-36-5
EINECS number	208-292-3
UNII number	7V515PI7F6
Compound type	Flavonoids
Molecular formula	C_15_H_10_O_5_
Molecular weight	270.24 g/mol
Form	Powder
Color	Pale yellow
Solubility	DMSO: 27 g/L
InChIKey	KZNIFHPLKGYRTM-UHFFFAOYSA-N
Density	1.548 g/cm^3^
pKa	6.53 ± 0.40 (Predicted)
Boiling point	555.5°C at 760 mmHg
Melting point	345°C–350°C
Flash point	217°C
Vapor pressure	0.0 ± 1.6 mmHg at 25°C
Refractivity	1.732
Polar surface area	90.90000
LogP	2.57680
Storage conditions	Sealed in dry, −20°C

## 3 The hepatoprotective effects and mechanisms of API in liver injury

### 3.1 Drug-induced liver injury

#### 3.1.1 Acetaminophen

Acetaminophen (APAP), also known as N-acetyl-p-aminophenol, is a widely used analgesic and antipyretic drug globally (Ishitsuka et al., 2020). However, improper or excessive use of APAP can lead to severe liver damage. Mitochondrial oxidative stress is considered a key mechanism in APAP-induced liver injury (Yan et al., 2018). Typically, APAP is metabolized in the liver and detoxified via glucuronidation and sulfation pathways (Yan et al., 2018). However, when consumed in excess, these pathways become saturated, leading to the formation of the toxic metabolite N-acetyl-p-benzoquinone imine (NAPQI) via the cytochrome P450 enzyme system (Jaeschke and Ramachandran, 2024). The accumulation of NAPQI within hepatocytes causes cellular damage and necrosis, ultimately leading to liver failure (Jaeschke and Ramachandran, 2024). Therefore, it is crucial to identify effective clinical antidotes to mitigate APAP-induced acute liver injury.

In 2013, Yang et al. (2013) first reported that API (100, 200 mg/kg) exhibited significant protective effects against APAP-induced acute liver injury in mice. This beneficial effect may be attributed to the increased activity of glutathione reductase (GR), which in turn elevated hepatic glutathione (GSH) levels (Yang et al., 2013). Similarly, Rašković et al. (2017) found that API (10 mg/kg) attenuated APAP-induced liver injury in rats by inhibiting lipid peroxidation levels and enhancing enzymatic antioxidant defense mechanisms. Subsequently, Zhao et al. (2020) and Zhang et al. (2020) further confirmed the ameliorative effects of API in APAP-induced liver injury through both *in vivo* and *in vitro* experiments. On the one hand, API can alleviate APAP-induced liver injury by promoting autophagy through the regulation of the SIRT1-p53 axis, thus reducing inflammation and oxidative stress (Zhao et al., 2020). On the other hand, API can reverse APAP-induced liver injury by activating the AMPK/GSK-3β signaling pathway, promoting CPT1A activity, and activating the Nrf2 antioxidant pathway (Zhang et al., 2020).

#### 3.1.2 Methotrexate

Methotrexate (MTX) is an antimetabolite drug widely used in the treatment of cancer, autoimmune diseases, and chronic inflammatory conditions (Mahmoud et al., 2023). However, high doses or prolonged use of MTX can lead to liver injury through various mechanisms, including inflammatory responses, oxidative stress, mitochondrial dysfunction, and metabolic disturbances, making hepatotoxicity one of the main side effects in clinical applications (Shetty et al., 2017). Therefore, to enhance the safety of MTX therapy, it is essential to adjust the dosage based on the patient’s specific condition or consider adjunctive treatments.

Studies have shown that pretreatment with API (20 mg/kg) significantly improves the levels of hepatic antioxidant markers (GSH, CAT, GPX and SOD) in mice with MTX-induced liver injury, and reduces levels of liver function indicators (AST, ALT and ALP) and inflammatory factors (TNF-α and IL-1β) (Goudarzi et al., 2021). Another study also found that pretreatment with API (3 mg/kg) alleviated MTX-induced liver injury by restoring the antioxidant defense system (MDA, SOD, CAT, GSH-Px and GSH), and reducing the expression of apoptotic factors (Caspase-3) and inflammatory factors (CRP, G-CSF and iNOS) (Sahindokuyucu-Kocasari et al., 2021). In summary, API can prevent MTX-induced liver injury by reducing inflammation and oxidative stress.

#### 3.1.3 Cyclophosphamide

Cyclophosphamide (CYC) is an alkylating agent widely used in the treatment of cancer and autoimmune diseases (Ahlmann and Hempel, 2016). However, its clinical use is limited by dose-dependent toxicity, particularly hepatotoxicity (Emadi et al., 2009). CYC-induced liver injury is mainly mediated by its metabolites, phosphoramide mustard and acrolein, which can trigger oxidative stress, lipid peroxidation, and inflammatory responses, leading to hepatocyte apoptosis and necrosis, thereby exacerbating liver injury (Moore, 1991; Üsküdar Cansu et al., 2019). Therefore, in clinical practice, to reduce CYC-induced liver injury, it is necessary to regularly monitor liver function and consider the use of antioxidants or other protective agents for adjunctive therapy. Fortunately, Al-Amarat et al. (2022) have shown that pretreatment with API (20 and 40 mg/kg) significantly ameliorates CYC-induced liver injury in rats, reducing levels of ALT, AST, ALP, and LDH, and inhibiting the expression of ROS, LPO, NF-κB, pro-inflammatory mediators (TNF-α, IL-6 and iNOS), and apoptotic markers (Bax and Caspase-3). Mechanistically, the hepatoprotective effect of API is associated with the upregulation of the Nrf2/HO-1 signaling pathway and enhanced antioxidant defense (Al-Amarat et al., 2022).

### 3.2 Chemical liver injury

#### 3.2.1 Furan

Furan (Fu) is a highly hepatotoxic industrial chemical and food contaminant, with liver injury primarily mediated by oxidative stress and inflammatory responses induced by its metabolites (Batool et al., 2021). In the liver, Fu is metabolized by the cytochrome P450 enzyme system to form reactive intermediates that react with cellular biomolecules, leading to lipid peroxidation, protein, and DNA damage (de Conti et al., 2017). Interestingly, Wang et al. (2014) demonstrated that API (5, 10, and 20 mg/kg) effectively alleviated Fu-induced liver and kidney injury. On the one hand, API increased the activity of GSH, GST and SOD, and reduced the levels of MPO, MDA and ROS (Wang et al., 2014). On the other hand, API decreased levels of pro-inflammatory cytokines IL-1β, IL-6 and TNF-α, and increased the level of the anti-inflammatory cytokine IL-10 (Wang et al., 2014). Mechanistically, the protective effects of API are mainly attributed to its excellent free radical scavenging ability and inhibition of lipid peroxidation (Wang et al., 2014). Therefore, consuming API-rich foods or using it as a dietary supplement could be significant for individuals at risk of Fu toxicity.

#### 3.2.2 Carbon tetrachloride

Carbon tetrachloride (CCl_4_) is a potent hepatotoxic substance widely used in experimental models to induce liver injury, liver fibrosis, cirrhosis, and liver cancer (Scholten et al., 2015). In the body, CCl_4_ is metabolized by microsomal enzymes in hepatocytes into trichloromethyl radicals (·CCl_3_) and trichloromethyl peroxy radicals (·CCl_3_O_2_) (Weber et al., 2003). These reactive intermediates can react with cellular membrane lipids, proteins, and DNA, leading to lipid peroxidation, membrane damage, and DNA breaks (Unsal et al., 2021). In a mouse model of CCl_4_-induced acute liver injury, API (50, 100, 200 mg/kg) significantly alleviated lipid peroxidation, as evidenced by increased levels of SOD, GSH, GSH-Px and CAT, and decreased levels of MDA (Yue et al., 2020). Additionally, API reduced the inflammatory response by decreasing the secretion of TNF-α and IL-6, and increasing the secretion of IL-10 (Yue et al., 2020). Similarly, in an H_2_O_2_-induced HepG2 cell model, API (10, 20, and 40 µM) reversed the imbalance between SOD, GSH activity and excessive ROS, and reduced the expression of IL-6 and TNF-α (Yue et al., 2020). In summary, API may alleviate liver injury by inhibiting the non-canonical NF-κB pathway, thereby reducing inflammation and oxidative stress (Yue et al., 2020).

#### 3.2.3 Di (2-ethylhexyl) phthalate

Di (2-ethylhexyl) phthalate (DEHP) is a widely used plasticizer commonly found in various plastic products (Zhang X. et al., 2022). Unfortunately, DEHP exhibits significant hepatotoxicity and can infiltrate daily life through various routes, posing potential harm to the liver (Martínez-Razo et al., 2021). In a DEHP-induced AML12 cell model, DEHP induced ferroptosis by increasing ROS levels, disrupting iron homeostasis, triggering lipid peroxidation, and regulating the expression of ferroptosis-related genes (Han et al., 2022). Notably, API (2.7 × 10^−3^ μg/mL) significantly mitigated these adverse changes by modulating GPX4 activity and inhibiting intracellular iron accumulation (Han et al., 2022). These results suggest that API may serve as a potential detoxifying agent with protective effects against DEHP-induced liver injury.

#### 3.2.4 Alcohol

Chronic or excessive alcohol consumption can significantly damage the liver, leading to the development of ALD (Mackowiak et al., 2024). Currently, alcohol-induced liver injury is one of the leading causes of liver diseases worldwide, with mechanisms involving steatosis, lipid metabolism disorders, oxidative stress, inflammatory responses, apoptosis and fibrosis (Lackner and Tiniakos, 2019; Chen M. et al., 2023). Interestingly, Wang et al. (2017), Wang et al. (2018) were the first to demonstrate, through both *in vivo* and *in vitro* experiments, the protective effects of API against alcohol-induced liver injury. On the one hand, API (150, 300 mg/kg) alleviated alcohol-induced liver damage by promoting the degradation of acetaldehyde, a toxic metabolite of alcohol, improving PPARα-mediated lipid metabolism pathways, and regulating CYP2E1-mediated oxidative stress in the liver (Wang et al., 2017). On the other hand, API (6, 12, and 24 µM) modulated the protein expression of NF-κB and IκB-α, reducing the production of inflammatory cytokines and thereby mitigating alcohol-induced inflammatory damage to hepatocytes (Wang et al., 2018). These findings indicate that API may prevent or reverse alcohol-induced liver injury by acting as a GR activator and CYP2E1 inhibitor, providing a theoretical foundation for the development of new drugs to treat ALD.

#### 3.2.5 Lead acetate

Lead acetate (PbAc) is commonly used as a catalyst or reagent in organic synthesis but poses serious health and environmental hazards due to the toxicity of its lead ions (Lakka et al., 2023). Remarkably, in a rat model of PbAc-induced liver and kidney injury, API demonstrated significant protective effects (Fehaid et al., 2023). Specifically, API (20 mg/kg) inhibited oxidative reactions by upregulating the Nrf2/HO-1 signaling pathway and activating downstream antioxidant enzymes, while significantly reducing the production of pro-oxidants (Fehaid et al., 2023). Additionally, API lowered the expression of pro-inflammatory cytokines, effectively suppressing apoptosis in hepatocytes and renal cells induced by PbAc (Fehaid et al., 2023). Overall, due to its potent antioxidant, anti-inflammatory, and anti-apoptotic properties, API holds promise as a potential therapeutic agent to alleviate liver and kidney damage associated with lead exposure.

#### 3.2.6 N-nitrosodiethylamine

N-nitrosodiethylamine (NDEA) is a nitrosamine compound commonly found in industrial waste, tobacco smoke, and certain contaminated foods and drinking water, and it exhibits high hepatotoxicity and carcinogenicity (Janmeda et al., 2024). NDEA severely damages liver structure and function through multiple mechanisms, including metabolic activation, oxidative stress, inflammatory responses, apoptosis, and necrosis, which may ultimately lead to liver fibrosis and even liver cancer (Cahyani et al., 2021). Remarkably, Ali et al. (2014) demonstrated that API could mitigate NDEA-induced hepatotoxicity in rats. Specifically, API (10, 20, 40 mg/kg) dose-dependently reduced serum levels of ALT, AST, ALP and LDH, as well as the levels of lipid peroxidation and protein carbonyl content (Ali et al., 2014). Furthermore, comet assays revealed that API treatment significantly reduced DNA damage in hepatocytes, blood lymphocytes and bone marrow cells (Ali et al., 2014). In conclusion, API is not only protective against NDEA-induced liver injury, but also exhibits anti-genotoxic potential.

### 3.3 Immune-mediated liver injury

Lipopolysaccharide (LPS), an endotoxin found in the outer membrane of gram-negative bacteria, is widely used to establish models of immune-mediated liver injury (Pfalzgraff and Weindl, 2019). LPS-induced liver injury primarily occurs through the activation of Kupffer cells, leading to the release of large amounts of inflammatory mediators such as IL-1β, IL-6, and TNF-α, thereby triggering immune-related liver damage (Rathinam et al., 2019). In a mouse model of LPS-induced liver injury, API (100, 200 mg/kg) exhibited significant hepatoprotective effects by inhibiting NF-κB and MAPK signaling cascades, enhancing both enzymatic and non-enzymatic antioxidant levels, and effectively reducing oxidative stress, neutrophil infiltration and inflammatory responses (Berköz et al., 2021). These findings suggest that API is a potential therapeutic agent for preventing liver injury caused by endotoxemia and sepsis.

D-Galactosamine (D-GalN) is an amino sugar compound that specifically interferes with RNA and protein synthesis in hepatocytes, leading to apoptosis and necrosis, as well as severe hepatitis and liver dysfunction (Maes et al., 2016). When combined with LPS, D-GalN significantly enhances the toxic response in hepatocytes, making this combination commonly used to establish animal models of immune-mediated liver injury for screening hepatoprotective drugs and exploring the mechanisms of liver injury (Kim et al., 2014; Farghali et al., 2016). Zhou et al. (2017), Zhou et al. (2020) confirmed, through both *in vivo* and *in vitro* experiments, the protective effects of API against D-GalN/LPS-induced liver injury. Mechanistically, API (100, 200 mg/kg) enhanced antioxidant capacity by increasing Nrf2-mediated antioxidant enzyme levels, including SOD, CAT, GST and GR (Zhou et al., 2017). Additionally, API (2.5, 5, 10, and 20 µM) suppressed the NF-κB signaling pathway by increasing the expression of PPARγ protein, thereby reducing the production of inflammatory cytokines (Zhou et al., 2020).

### 3.4 Ischemia-reperfusion liver injury

Ischemia-reperfusion (I/R) liver injury refers to significant liver tissue damage that occurs during the process of temporary interruption and subsequent restoration of blood supply to the liver (Wu et al., 2018). The initial ischemic phase leads to hypoxia and metabolic stress, which are further exacerbated by oxidative stress, inflammatory responses, and apoptosis during the reperfusion phase, thereby aggravating liver injury (Jiménez-Castro et al., 2019; Bardallo et al., 2022). Kupffer cells in the liver play a key role in mediating inflammatory responses, while neutrophil activation and ROS production exacerbate liver tissue damage (Jiménez-Castro et al., 2019). Unfortunately, I/R liver injury is a common clinical problem today, particularly during liver surgery and transplantation. Therefore, it is of great importance to find effective drugs to treat I/R liver injury.

Fortunately, Tsalkidou et al. (2014) and Tsaroucha et al. (2016) have confirmed that API exhibits significant protective effects in I/R liver injury. Specifically, API (15 mg/kg) reduced the activity of pro-apoptotic factors by modulating the expression of B-cell lymphoma-2 (Bcl-2) and Bcl-2-associated X (Bax) genes and decreased ICAM-1 levels, thereby reducing the release of inflammatory mediators and improving I/R -induced liver injury (Tsaroucha et al., 2016). Additionally, API (15 mg/kg) significantly reduced the expression levels of the Fas gene in hepatocytes during reperfusion (Tsalkidou et al., 2014). Notably, the Fas gene encodes the Fas receptor, a critical protein that initiates the apoptotic signaling pathway (Khan et al., 2017). This suggests that API may exert its protective effects against hepatic I/R injury by inhibiting the Fas/FasL-mediated apoptotic pathway (Tsalkidou et al., 2014).

### 3.5 Others

**FIGURE 3:**
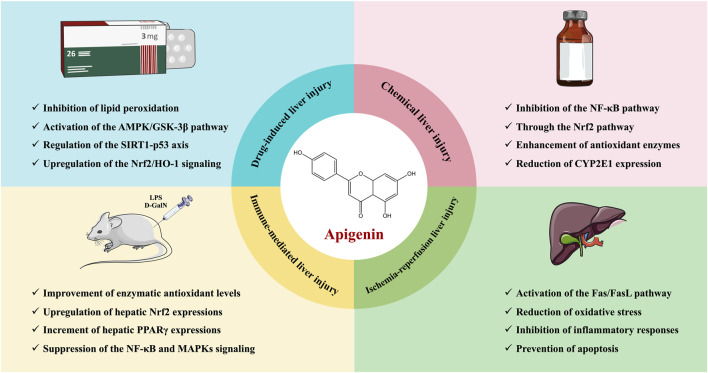
Schematic representation of the therapeutic role of API in liver injury.

Nickel oxide nanoparticles (NiONPs) are transition metal oxide nanomaterials widely used in medical sensors, battery electrodes, catalysts and other fields (Berhe and Gebreslassie, 2023). Since NiONPs induce oxidative stress and reduce antioxidant capacity, leading to liver damage, it is imperative to find ways to protect individuals working in related industries from occupational exposure-induced liver damage (Saquib et al., 2018). Interestingly, Ali et al. (2021) confirmed, through hematological, biochemical, histopathological and metal analyses, the protective effects of API (25 mg/kg) against NiONPs-induced liver injury in rats, with specific mechanisms including the inhibition of oxidative stress and inflammatory responses. Moreover, API (0.625, 1.25, and 2.5 µM) also demonstrated protective effects against palmitic acid (PA)-induced damage in HepG2 cells and primary mouse hepatocytes (Meng et al., 2022). The beneficial effects of API may be attributed to its ability to alleviate PA-induced pyroptosis by inhibiting NLRP3 inflammasome activation through the activation of autophagy (Meng et al., 2022). Notably, this study reveals a novel link between autophagy and pyroptosis, providing new insights and targets for the use of functional factors in food to alleviate liver damage. The hepatoprotective effects and mechanisms of API in liver injury are presented in Table 2. The therapeutic role of API in liver injury is shown schematically in Figure 3.

**TABLE 2:** The functions and molecular mechanisms of API in liver injury.

Models	Types	Routes	Dosage of administration	Molecular mechanisms	Years	References
APAP-induced acute liver injury in Kunming male mice	*In vivo*	p.o.	100, 200 mg/kg API for 7 days	Increment of hepatic GR activity	2013	Yang et al. (2013)
APAP-induced hepatotoxicity in albino Wistar male rats	*In vivo*	p.o.	10 mg/kg API for 6 days	Inhibits the level of lipid peroxidation and significantly increases the enzyme antioxidant defense mechanisms	2017	Rašković et al. (2017)
APAP-induced liver injury in C57BL/6 mice	*In vivo*	p.o.	20, 80 mg/kg API for 7 days	Regulation of the SIRT1-p53 axis	2020	Zhao et al. (2020)
APAP-treated LO2 cells	*In vitro*	N/A	5, 50 µM API for 24 h			
APAP-induced liver injury in C57BL/6 mice	*In vivo*	p.o.	20, 80 mg/kg API for 4 h	Activation of the AMPK/GSK-3β signaling pathway	2020	Zhang et al. (2020)
APAP-treated LO2 cells	*In vitro*	N/A	5, 25, 50 µM API for 15 min			
MTX-induced hepatotoxicity in Wistar male rats	*In vivo*	p.o.	20 mg/kg API for 9 days	Mitigation of oxidative stress and inflammation	2021	Goudarzi et al. (2021)
MTX-induced liver injury in CD-1 male mice	*In vivo*	i.p.	3 mg/kg API for 7 days	Attenuating oxidative stress and tissue injury markers, histopathological alterations, and apoptosis and inflammation	2021	Sahindokuyucu-Kocasari et al. (2021)
CYC-induced liver damage in Wistar male rats	*In vivo*	i.g.	20, 40 mg/kg API for 15 days	Upregulation of Nrf2/HO-1 signaling	2022	Al-Amarat et al. (2022)
Fu-induced toxicity in BALB/c male mice	*In vivo*	i.g.	5, 10, 20 mg/kg API for 14 days	By clearing free radicals and inhibiting lipid oxidation	2014	Wang et al. (2014)
CCl_4_-induced acute liver injury in ICR male mice	*In vivo*	i.g.	50, 100, 200 mg/kg API for 7 days	Inhibition of the non-canonical NF-κB pathway	2020	Yue et al. (2020)
H_2_O_2_-induced in HepG2 cells	*In vitro*	N/A	10, 20, 40 µM API for 24 h			
DEHP-treated AML12 cells	*In vitro*	N/A	2.7 × 10^−3^ μg/mL API for 24 h	By activating GPX4 and suppressing intracellular iron accumulation	2022	Han et al. (2022)
0.1 mM ethanol-treated BRL cells	*In vitro*	N/A	6, 12, 24 µM API for 2 h	Reduction of CYP2E1 expression, increment of antioxidant ability, and regulation of inflammatory gene expression	2018	Wang et al. (2018)
2 μg/mL LPS-treated BRL cells	*In vitro*	N/A	6, 12, 24 µM API for 2 h			
56% erguotou wine-induced alcoholic liver injury in Kunming male mice	*In vivo*	i.g.	150, 300 mg/kg API for 30 days	Regulating hepatic CYP2E1-mediated oxidative stress and PPARα-mediated lipogenic gene expression	2017	Wang et al. (2017)
PbAc-induced hepatorenal damage in Wistar albino male rats	*In vivo*	p.o.	20 mg/kg API for 7 days	Through the Nrf2 signaling pathway	2023	Fehaid et al. (2023)
NDEA-induced hepatotoxicity in Wistar male rats	*In vivo*	p.o.	10, 20, 40 mg/mL API for 21 days	Enhancement of antioxidant enzymes or direct scavenging of ethyl free radicals	2014	Ali et al. (2014)
LPS-induced acute liver injury in Balb/c male mice	*In vivo*	p.o.	100, 200 mg/kg API for 7 days	Suppressing the signaling cascades of NF-κB p65 and MAPKs and improving enzymatic and non-enzymatic antioxidant levels	2021	Berköz et al. (2021)
D-GalN/LPS-induced acute liver injury in ICR male mice	*In vivo*	i.g.	100, 200 mg/kg API for 7 days	Upregulation of hepatic Nrf2 and PPARγ expressions	2017	Zhou et al. (2017)
D-GalN/LPS-stimulated rat BRL cells	*In vitro*	N/A	2.5, 5, 10, 20 µM API for 2 h	Increment of hepatic Nrf2 and PPARγ expressions	2020	Zhou et al. (2020)
I/R injury in Wistar-type rats	*In vivo*	i.p.	15 mg/kg API for 60/120/240 min	Through the Fas/FasL mediated pathway of apoptosis	2014	Tsalkidou et al. (2014)
I/R injury in Wistar-type rats	*In vivo*	i.p.	15 mg/kg API for 60/120/240 min	Suppression of inflammation, oxidative stress and apoptosis	2016	Tsaroucha et al. (2016)
NiONPs-induced toxicity in Wistar male rats	*In vivo*	p.o.	25 mg/kg API for 28 days	Possibly through its anti-inflammatory and antioxidant effects	2021	Ali et al. (2021)
PA-treated HepG2 cells	*In vitro*	N/A	0.625, 1.25, 2.5 µM API for 3 h	Inhibition of NLRP3 inflammasome activation by activation of autophagy	2022	Meng et al. (2022)
PA-treated primary mouse hepatic cells	*In vitro*	N/A	0.625, 1.25, 2.5 µM API for 3 h			

## 4 The hepatoprotective effects and mechanisms of API in NAFLD/NASH

### 4.1 PPAR signaling pathway

PPARs are well-known for their critical role in the pathogenesis of NAFLD and NASH. Specifically, PPARα ameliorates hepatic steatosis by promoting fatty acid β-oxidation and reducing hepatic lipid accumulation (Khan et al., 2019). PPARγ plays a key role in adipocyte differentiation, lipid storage, and anti-inflammatory responses, and it reduces liver inflammation and fibrosis by modulating macrophage polarization (Chen H. et al., 2023). Additionally, PPARδ is significant in regulating insulin sensitivity and systemic lipid metabolism (Qiu et al., 2023). Therefore, therapeutic strategies targeting PPARs have shown potential efficacy and promising prospects in clinically improving NAFLD/NASH.

Interestingly, Shi et al. (2015) demonstrated that API alleviates hepatic steatosis and inflammatory necrosis in a high-fat diet (HFD)-induced NASH rat model by improving insulin resistance and glucose-lipid metabolism. The molecular mechanism involves the regulation of PPARα and PPARγ expression, suggesting that API might act as a dual agonist of PPARα/PPARγ (Shi et al., 2015). It combines the triglyceride-lowering effects of fibrates (PPARα agonists) with the insulin-sensitizing and lipid-regulating functions of thiazolidinediones (PPARγ agonists). Similarly, Hsu et al. (2021) found that API (20 µM) significantly increased the expression of proteins associated with the β-oxidation pathway, including PPARα, in oleic acid (OA)-treated Huh7 cells, thereby reducing intracellular lipid levels. Mechanistically, API may improve hepatic lipid accumulation by activating the autophagy-mitochondrial pathway (Hsu et al., 2021). These findings provide new insights into the potential of API in modulating dysregulated hepatic lipid metabolism.

Notably, PPARγ is also considered a major regulator of macrophage polarization (Wang et al., 2021b). Subsequent studies have shown that API (7.5 µM) significantly reverses M1 to M2 macrophage polarization in HFD and ob/ob mouse models, reducing inflammatory cell infiltration in the liver and lowering the secretion of pro-inflammatory cytokines, thereby alleviating obesity-associated inflammation (Feng et al., 2016). The underlying mechanism may involve the modulation of p65/PPARγ complex localization, altering macrophage functional polarization (Feng et al., 2016). Importantly, unlike the clinically used thiazolidinedione drug rosiglitazone, API does not cause side effects such as weight gain and osteoporosis (Feng et al., 2016). These results suggest that API may be a potential candidate for treating obesity-associated inflammation in the future.

### 4.2 Nrf2 signaling pathway

Oxidative stress is recognized as a key mechanism in the pathogenesis and progression of NAFLD/NASH (Chen et al., 2020). Numerous studies have shown that the Nrf2 signaling pathway plays a protective role in NAFLD (Xu et al., 2018; Bathish et al., 2022). Specifically, Nrf2 enhances cellular antioxidant defense by regulating the expression of a series of antioxidant enzymes, such as SOD, CAT and GST, thereby reducing the impact of oxidative damage on the liver (Xu et al., 2018). Additionally, Nrf2 can regulate inflammatory responses and lipid metabolism by inhibiting NF-κB activation and reducing the release of inflammatory mediators, thus lowering hepatic inflammation and lipid accumulation, ultimately helping to block the progression of NAFLD/NASH (Bathish et al., 2022). Therefore, activating the Nrf2 signaling pathway may represent a potential strategy for treating NAFLD/NASH.

Zhou (2016) and Feng et al. (2017) found that API (30 mg/kg) significantly inhibited lipid droplet formation in the liver, increased the expression of antioxidant genes, and suppressed the expression of lipogenic genes in an HFD-induced NAFLD mouse model. These effects were achieved by regulating the expression of downstream oxidative stress-related genes through the Nrf2 signaling pathway (Zhou, 2016; Feng et al., 2017). Further experiments demonstrated that API’s regulation of PPARγ target genes depended on Nrf2 activation, while Nrf2 activation offset the activation of PPARγ by API (Zhou, 2016; Feng et al., 2017). Moreover, Yang M. et al. (2018) showed that API (50 mg/kg) prevented multiple adverse metabolic effects induced by high fructose, including insulin resistance, dyslipidemia, and liver injury. The mechanism may involve the interaction between API and Keap1, blocking the binding of Keap1 to Nrf2, thereby increasing the transcriptional expression of Nrf2-targeted antioxidant genes (Yang M. et al., 2018). Collectively, these studies suggest that API, through the modulation of the Nrf2 signaling pathway, holds significant potential for mitigating NAFLD and improving metabolic syndrome.

### 4.3 NLRP3 signaling pathway

The NLRP3 inflammasome is a multiprotein complex whose activation triggers hepatic inflammatory responses, promoting hepatocyte injury and lipid accumulation (Fu and Wu, 2023). Specifically, upon activation, NLRP3 initiates downstream Caspase-1, leading to the release of pro-inflammatory cytokines such as IL-1β and IL-18 (Huang et al., 2021). The release of these cytokines not only exacerbates the inflammatory response in the liver, but also promotes hepatocyte injury and fibrosis, furthering the progression of NAFLD to NASH (de Carvalho Ribeiro and Szabo, 2022). Therefore, inhibiting the activity of the NLRP3 signaling pathway is considered a potential strategy for treating NAFLD/NASH.

Interestingly, Lv et al. (2019) reported that API (50 mg/kg) ameliorated hepatic lipid accumulation and inflammation, alleviating HFD-induced NAFLD. This beneficial effect may be attributed in part to the modulation of xanthine oxidase (XO) by API, which further inhibits the activation of NLRP3 inflammatory vesicles and the release of the inflammatory cytokines IL-1β and IL-18 (Lv et al., 2019). Similarly, Lu et al. (2023) confirmed through *in vitro* and *in vivo* experiments that API (5, 50 mg/kg) alleviated NAFLD by inhibiting the NLRP3 inflammasome. Furthermore, Meng et al. (2023) demonstrated that API (25, 50, and 100 mg/kg) reduced HFD-induced liver injury by activating mitophagy. Mechanistically, API may inhibit NLRP3 inflammasome activation by clearing ROS production, reducing lysosomal membrane permeability and cathepsin B (CTSB) release (Meng et al., 2023).

### 4.4 AMPK/SREBP signaling pathway

AMPK, as a cellular energy sensor, reduces hepatic lipid synthesis by inhibiting SREBP activation, thereby ameliorating NAFLD (Smith et al., 2016). Specifically, upon activation, AMPK inhibits the expression of fatty acid synthase (FAS) and acetyl-CoA carboxylase, enzymes that are induced by SREBP for lipid synthesis (Fang et al., 2022). Additionally, AMPK alleviates hepatic steatosis and inflammation by promoting fatty acid oxidation and reducing lipid accumulation (Fang et al., 2022). Therefore, the regulation of the AMPK/SREBP pathway is importance for the prevention and treatment of NAFLD.

In PA-induced HepG2 cells, API (10, 20, and 40 µM) significantly reduced total cholesterol (TC) and triglyceride (TG) levels (Lu et al., 2019). Moreover, API increased AMPK activity in a concentration-dependent manner while decreasing the expression of 3-hydroxy-3-methylglutaryl-CoA reductase, FAS and SREBP-1/2 (Lu et al., 2019). Mechanistically, API improves lipid metabolism by activating the AMPK/SREBP signaling pathway, thereby reducing excessive hepatic lipid accumulation (Lu et al., 2019).

### 4.5 JAK2-STAT3 signaling pathway

Studies have shown that excessive activation of the JAK2-STAT3 pathway leads to lipid accumulation and insulin resistance in hepatocytes, further exacerbating hepatic inflammation and steatosis (Chen B. et al., 2023). Conversely, inhibiting the activity of this signaling pathway can reduce hepatic inflammation and steatosis, thereby improving NAFLD symptoms (Huang et al., 2022). Therefore, the JAK2-STAT3 signaling pathway is considered a potential target for treating NAFLD, and regulating this pathway may effectively intervene and slow the disease’s progression.

Interestingly, Ma (Ma, 2019) explored the role and mechanism of API in anti-NAFLD through *in vitro* PA-induced LO2 cell models and *in vivo* HFD-induced NAFLD mouse models. The results of the cell experiments showed that API (25, 50, and 100 µM) improved lipid accumulation to varying degrees compared with the model group (Ma, 2019). The results of animal experiments further demonstrated that API (200 mg/kg) significantly ameliorated hepatic steatosis and reduced serum levels of TG, TC, GLU, LDL, AST, and ALT (Ma, 2019). Mechanistically, API may exert its anti-NAFLD effects by inhibiting the JAK2/STAT3 signaling pathway (Ma, 2019).

### 4.6 Others

Furthermore, in HFD-induced mice, API (0.005% w/w) reduced plasma levels of free fatty acids, total cholesterol, apolipoprotein B, and liver function markers, while ameliorating hepatic steatosis and liver hypertrophy (Jung et al., 2016). This beneficial effect is partly attributed to API’s regulation of hepatic metabolic and transcriptional responses, including upregulation of genes related to fatty acid oxidation, the tricarboxylic acid cycle, oxidative phosphorylation, the electron transport chain, and cholesterol homeostasis, as well as downregulation of genes related to lipolysis and lipogenesis (Jung et al., 2016). It is worth noting that Fetuin-A, a glycoprotein synthesized and secreted by hepatocytes into the bloodstream, can impede insulin signaling activation by binding to the extracellular region of the insulin receptor, leading to insulin resistance (Lanthier et al., 2022). Interestingly, Hsu et al. (2024) reported that API (20 mg/kg) improved hepatic insulin resistance by targeting Fetuin-A in HFD-induced mice. This new finding expands the biomedical significance of API in the prevention and treatment of HFD-induced metabolic disorders. The hepatoprotective effects and mechanisms of API in NAFLD/NASH are presented in Table 3.

**TABLE 3:** The functions and molecular mechanisms of API in NAFLD/NASH.

Models	Types	Routes	Dosage of administration	Molecular mechanisms	Years	References
Sprague-Dawley rats fed HFD	*In vivo*	i.g.	15, 30, 60 mg/kg API for 8 weeks	Promotion of PPARα and PPARγ expression	2015	Shi et al. (2015)
C57BL/6J male mice fed HFD	*In vivo*	i.p.	10, 30, 50 mg/kg API for 3 weeks	Modification of macrophage functional polarization via regulation of the location of p65/PPARγ	2016	Feng et al. (2016)
C57BL/6J ob/ob male mice	*In vivo*	i.p.	30 mg/kg API for 3 weeks			
LPS/IL-4-treated ANA-1 cells and RAW264.7 cells	*In vitro*	N/A	7.5 µM API for 24 h			
OA-treated Huh7 cells	*In vitro*	N/A	2.5, 5, 10, 20 µM API for 18 h	By activating the autophagic mitochondrial pathway	2021	Hsu et al. (2021)
OA-treated Huh7.5 cells	*In vitro*	N/A	20 µM API for 3/6/9/18 h			
OA-treated HepG2 cells	*In vitro*	N/A	20 µM API for 18 h			
OA-treated AML12 cells	*In vitro*	N/A	20 µM API for 18 h			
C57BL/6 mice fed HFD	*In vivo*	i.p.	30 mg/kg API for 3 weeks	Promotion of antioxidant gene expression through activation of Nrf2	2016	Zhou (2016)
Free fatty acids-treated Hep1-6 cells	*In vitro*	N/A	1, 5, 10, 20 µM API for 24 h			
C57BL/6J male mice fed HFD	*In vivo*	i.p.	30 mg/kg API for 3 weeks	Regulating hepatocyte lipid metabolism and oxidative stress via Nrf2 activation	2017	Feng et al. (2017)
Kunming male mice fed high fructose	*In vivo*	i.g.	50 mg/kg API for 4 weeks	Through the Keap1-Nrf2 pathway	2018	Yang M. et al. (2018)
C57BL/6J male mice fed HFD	*In vivo*	i.g.	50 mg/kg API for 4 weeks	Regulation of the XO/NLRP3 pathways	2019	Lv et al. (2019)
*Ldlr* ^*−/−*^ male mice fed HFD	*In vivo*	i.g.	5, 50 mg/kg API for 8 weeks	Inhibition of inflammation and NLRP3 activation	2023	Lu et al. (2023)
NLRP3^*−/−*^ Ldlr^*−/−*^ male mice fed HFD	*In vivo*	i.g.	50 mg/kg API for 8 weeks			
LPS and OA-stimulated HepG2 cells	*In vitro*	N/A	25, 50 µM API for 24 h			
C57BL/6 male mice fed HFD	*In vivo*	i.g.	25, 50, 100 mg/kg API for 4 weeks	Through the mitophagy-ROS-CTSB-NLRP3 pathway.	2023	Meng et al. (2023)
PA-treated AML-12 cells	*In vitro*	N/A	0.625, 1.5, 2.5 µM API for 24 h			
PA-treated HepG2 cells	*In vitro*	N/A	10, 20, 40 µM API for 24 h	Activation of the intracellular AMPK/SREBP signaling pathway	2019	Lu et al. (2019)
PA-treated LO2 cells	*In vitro*	N/A	25, 50, 100 µM API for 24 h	Inhibition of JAK2/STAT3-mediated signaling pathway	2019	Ma (2019)
C57BL/6 male mice fed HFD	*In vivo*	i.g.	200 mg/kg API for 16 weeks			
C57BL/6JOlaHsd male mice fed HFD	*In vivo*	p.o.	500 mg/kg API for 12 weeks	Reduction of hepatic *Cyp2b9* transcript levels	2015	Hoek-van den Hil et al. (2015)
C57BL/6 male mice fed HFD	*In vivo*	p.o.	0.005% (w/w) API for 16 weeks	Modulation of metabolic and transcriptional profiles	2016	Jung et al. (2016)
PA-treated Huh7 cells	*In vitro*	N/A	5, 10, 20 µM API for 12 h	Regulation of fetuin-A gene expression and protein phosphorylation of fetuin-A, and the interaction between fetuin-A and insulin receptor	2024	Hsu et al. (2024)
C57BL/6 male mice fed HFD	*In vivo*	p.o.	20 mg/kg API for 12 weeks			

## 5 The hepatoprotective effects and mechanisms of API in liver fibrosis

Liver fibrosis is a pathological process induced by chronic liver injury, characterized by excessive deposition of extracellular matrix (ECM) within the liver (Aydın and Akçalı, 2018). It is commonly caused by chronic liver diseases such as viral hepatitis, ALD, NAFLD, and cholestatic liver diseases (Parola and Pinzani, 2019). During this process, hepatic stellate cells (HSCs) are activated and produce large amounts of ECM components, leading to the gradual destruction of liver structure and function (Higashi et al., 2017). The progression of liver fibrosis can range from reversible to irreversible. In the early stages, fibrosis can be significantly reversed through the removal of the causative factors or therapeutic interventions, while in the late stages, it may progress to irreversible cirrhosis, ultimately leading to liver failure and HCC (Parola and Pinzani, 2019). Current treatment strategies include the removal of the causative factors and the development of antifibrotic drugs, with research focusing on inhibiting HSC activation, reducing ECM production, promoting ECM degradation, and modulating related cellular signaling pathways (Roehlen et al., 2020; Zhang D. et al., 2022).

Fortunately, Ji et al. (2021) confirmed the protective effects of API against liver fibrosis. API effectively alleviates CCl_4_-and bile duct ligation (BDL)-induced liver fibrosis by reducing liver enzyme levels, inhibiting ECM production, and regulating the balance between matrix metalloproteinase 2 (MMP2) and tissue inhibitors of metalloproteinase 1 (TIMP1) (Ji et al., 2021). Mechanistically, API may exert its antifibrotic effects by inhibiting the activation of HSCs and autophagy through the TGF-β1/Smad3 and p38/PPARα signaling pathways (Ji et al., 2021). Similarly, Melaibari et al. (2023) and Sun et al. (2024) also evaluated the antifibrotic efficacy of API in CCl_4_-induced liver fibrosis mouse models. On the one hand, API significantly mitigates oxidative stress, inflammation, and pathological angiogenesis by restoring GSH levels and CAT activity, reducing lipid peroxidation, inhibiting the expression of pro-inflammatory cytokines IL-1β, IL-6 and TNF-α, and decreasing the expression of pro-angiogenic factors VEGF and CD34 (Melaibari et al., 2023). On the other hand, API exerts its remarkable hepatoprotective effects by inhibiting HSC activation through pathways such as the regulation of the EGFR-MEK1/2-ERK1/2 signaling pathway, inhibition of the PKM2-HIF-1α pathway, and mediation of oxidative stress (Sun et al., 2024).

Cholestasis is a disease caused by disturbances in bile secretion and metabolism, leading to the accumulation of toxic bile acids that can cause damage to hepatocytes and the entire body (Méndez-Sánchez et al., 2023). If left untreated, cholestasis can gradually progress to liver fibrosis and even cirrhosis (Zeng et al., 2023). Notably, studies have shown that API has protective effects against DDC-induced cholestatic liver disease (Zheng et al., 2021). Mechanistically, API enhances the antioxidant capacity of hepatocytes by upregulating the activity of antioxidant enzymes, and alleviates DDC-induced liver injury and fibrosis in mice by inhibiting the TLR4/NF-κB/TNF-α inflammatory signaling pathway and upregulating FXR expression to regulate bile acid metabolism (Zheng et al., 2021). These findings suggest that API holds potential as a therapeutic agent for cholestatic liver diseases, such as primary biliary cholangitis and primary sclerosing cholangitis.

In recent years, the application of transcriptomics in liver fibrosis research has significantly advanced this field. Through comprehensive analysis of gene expression profiles in fibrotic liver tissues, scientists have been able to uncover key genes and signaling pathways involved in the fibrotic process (Zhang and Zhang, 2020). In 2017, Hicks et al. (2017) identified API as a potential antifibrotic agent by querying transcriptomic databases. Subsequent studies demonstrated that API (2.5, 10, and 40 µM) reduces collagen I expression in a dose-dependent manner in the human HSC line TWNT-4 (Hicks et al., 2017). Mechanistically, API may exert its antifibrotic effects by regulating C1QTNF2, a secretory adipocytokine with hepatic metabolic effects (Hicks et al., 2017). Additionally, in a CCl_4_-induced liver fibrosis rat model, API (150, 300, and 600 mg/kg) significantly reduced various biochemical parameters and alleviated liver fibrosis and inflammation (Qiao et al., 2020). Further transcriptomic and proteomic analyses revealed that the protective effects of API on liver fibrosis in rats might be achieved through VEGF-mediated FAK phosphorylation and multiple signaling pathways, including MAPKs, PI3K/Akt, HIF-1, ROS and eNOS (Qiao et al., 2020).

Furthermore, Duan et al. (2024) established a transcription-based drug screening system and identified API from 283 natural compounds due to its ability to reverse gene expression patterns associated with PANoptosis and type I interferon (IFN-I) signaling. Subsequently, in BDL, Abcb4^−/−^ and DDC-fed mouse models, API (25, 50 mg/kg) effectively alleviated liver injury, inflammation, and fibrosis, protected cholangiocytes from bile acid-induced PANoptosis, and inhibited IFN-I-induced inflammatory responses (Duan et al., 2024). Notably, this study not only demonstrated the pathogenic role of PANoptosis and IFN-I signaling in cholestatic liver fibrosis but also revealed a novel anti-inflammatory mechanism of API, making it a promising candidate for the treatment of cholestatic liver fibrosis (Duan et al., 2024). The hepatoprotective effects and mechanisms of API in liver fibrosis are presented in Table 4.

**TABLE 4:** The functions and molecular mechanisms of API in liver fibrosis.

Models	Types	Routes	Dosage of administration	Molecular mechanisms	Years	References
TWNT-4 cells	*In vitro*	N/A	2.5, 10, 40 µM API for 24 h	Modulation of C1QTNF2 expression	2017	Hicks et al. (2017)
CCl_4_-induced liver fibrosis in Wistar male rats	*In vivo*	i.g.	150, 300, 600 mg/kg API for 5 weeks	Through the MAPKs, PI3K/Akt, HIF-1, ROS, and eNOS pathways	2020	Qiao et al. (2020)
Hydrogen peroxide-treated TFK-1 cells	*In vitro*	N/A	0.1, 1, 5, 10 µM API for 24 h	Inhibition of TLR4/NF-κB/TNF-α inflammatory signaling pathway and upregulation of FXR expression regulates bile metabolic homeostasis	2021	Zheng et al. (2021)
Lithocholic acid-treated Huh7 cells	*In vitro*	N/A	0.1, 1, 5, 10 µM API for 12 h			
TGF-β-treated LX2 cells	*In vitro*	N/A	1, 5, 10 µM API for 12 h			
DDC-induced liver fibrosiss in C57BL/6J male mice	*In vivo*	i.g.	30 mg/kg API for 4 weeks			
LX-2 cells	*In vitro*	N/A	20, 40, 60 µM API for 24 h	Inhibition of the TGF-β1/Smad3 and p38/PPARα pathways	2021	Ji et al. (2021)
CCl_4_-induced liver fibrosis in C57BL/6 male mice	*In vivo*	i.g.	20, 40 mg/kg API for 8 weeks (three times a week)			
BDL-induced liver fibrosis in C57BL/6 male mice	*In vivo*	i.g.	20, 40 mg/kg API for 14 days			
CCl_4_-induced liver fibrosis in Swiss albino male mice	*In vivo*	i.g.	2, 20 mg/kg API for 6 weeks (three times weekly)	Modulation of oxidative stress, inflammation, and fibrogenesis	2023	Melaibari et al. (2023)
BDL-induced liver fibrosis in C57BL/6J male mice	*In vivo*	i.g.	25, 50 mg/kg API for 1 week	Inhibition of PANoptosis and following IFN-I responses	2024	Duan et al. (2024)
Abcb4^−/−^ male mice	*In vivo*	i.g.	25, 50 mg/kg API for 4 weeks			
DCC-induce cholestatic liver fibrosis in Wistar male rats	*In vivo*	i.g.	25, 50 mg/kg API for 3 weeks			
CDCA-stimulated HIBEC cells	*In vitro*	N/A	2.5, 5, 10 µM API for 24 h			
CCl_4_-induced liver fibrosis in C57BL/6J male mice	*In vivo*	i.g.	10, 20, 40 mg/kg API for 6 weeks	Regulating EGFR-MEK1/2-ERK1/2 signaling, inhibiting the PKM2-HIF-1α pathway and mediating oxidative stress	2024	Sun et al. (2024)

## 6 The hepatoprotective effects and mechanisms of API in liver cancer

### 6.1 Monotherapy

#### 6.1.1 Inhibition of proliferation of HCC cells

In the treatment of HCC, inhibiting the proliferation of cancer cells is a crucial step. Numerous studies have demonstrated that API effectively inhibits the proliferation of various HCC cell lines (Chiang et al., 2006; Cui et al., 2018). For instance, API significantly suppresses the proliferation of HepG2 cells, Huh-7 cells, MHCC97 cells, MHCC97 cell-derived second-generation sphere cells, Hep3B cells, and PLC/PRF/5 cells, with IC_50_ values of 11.0 μg/mL, 12.0 μg/mL, 43.2 ± 2.3 μM, 18.4 ± 1.6 μM, 22.16 ± 0.67 μg/mL, and 22.55 ± 1.42 μg/mL, respectively (Chiang et al., 2006; Bhattacharya et al., 2018; Cui et al., 2018). Similarly, Chen et al. (2015) and Li et al. (2017) confirmed that API significantly inhibits the proliferation of HepG2 cells in a time- and concentration-dependent manner.

Notably, API has the potential to inhibit HCC cell proliferation by regulating the cell cycle. Wang (Wang, 2020) revealed that API (5, 10, and 20 mg/L) effectively arrests the cell cycle of Huh-7 cells at the G2/M phase, and this arresting effect is significantly enhanced with increasing concentrations of API. Further microRNA (miRNA) transcriptome sequencing analysis confirmed that API may mediate the cell cycle arrest of Huh-7 cells by regulating the differential expression of miRNAs (Wang, 2020). MED28, a subunit of the transcriptional activator, has been shown to play a key role in the occurrence and development of various malignancies (Cho et al., 2019). Interestingly, Chou et al. (2024) demonstrated that API could regulate the MED28/mTOR signaling pathway, leading to cell cycle arrest, affecting the nuclear translocation of SREBP-1, reducing lipid accumulation and ultimately inhibiting the development of HCC.

Research has shown that the high expression of KDM1A promotes the proliferation and invasion of HCC cells and is associated with tumor aggressiveness and poor prognosis (Ismail et al., 2018). Therefore, KDM1A is considered a potential target in HCC treatment. Zhang (2020) indicated that API (50, 100 µM) can inhibit the proliferation and growth of HCC cells by targeting KDM1A to regulate lipid metabolism. Moreover, the abnormal overexpression of long non-coding RNA H19 is closely related to the occurrence and progression of HCC (Zhang et al., 2019). Interestingly, Pan et al. (2021) revealed that API (50 mg/kg) can downregulate H19 and reduce the expression of β-catenin, leading to the inactivation of the Wnt/β-catenin signaling pathway, thereby inhibiting tumor growth. This study not only provides a new mechanism of tumor suppression mediated by API but also suggests that API may be a promising candidate drug for cancer patients.

#### 6.1.2 Induction of apoptosis of HCC cells

Apoptosis, a form of programmed cell death, plays a critical role in maintaining tissue health and stability by eliminating abnormal or damaged cells (Singh and Lim, 2022). In the treatment of HCC, inducing apoptosis of tumor cells is a major strategy, as it effectively reduces the number of cancer cells, inhibiting tumor growth and metastasis (Anwanwan et al., 2020). It is well known that Bcl-2, as a member of the apoptosis protein family, plays a role in inhibiting apoptosis, while Bax suppresses tumor growth by promoting apoptosis (Hafezi and Rahmani, 2021). Interestingly, Wang (Wang, 2020) demonstrated that API (50 μg/d) significantly reduces the expression of Bcl-2 while increasing the expression of Bax, thereby inducing apoptosis. Additionally, Khan and Sultana (2006) showed that API (25, 50 µM) can induce apoptosis in HepG2 cells by activating the Caspase pathway and promoting the production of TNF-α and IFN-γ. Similarly, Seydi et al. (2016) also confirmed that API (10, 20, and 40 µM) selectively induces apoptosis in HCC cells and inhibits tumor growth by directly targeting mitochondria.

API can also induce apoptosis of HCC cells by regulating multiple signaling pathways. The p53-WAF/p21 pathway is an important cell signaling pathway that mainly regulates the cell cycle and maintains genomic stability (Engeland, 2022). Interestingly, Chiang et al. (2006) revealed that API (4, 8, and 16 mg/mL) induces apoptosis in HepG2 cells, significantly increases p53 accumulation, and elevates p21/WAF1 levels. Mechanistically, API-induced apoptosis in HCC cells may be mediated through the p53-WAF/p21 pathway (Chiang et al., 2006). It is worth mentioning that API’s growth-inhibitory effect on HepG2 cells is comparable to the efficacy of the clinically used anti-HCC drug 5-fluorouracil.

Loss or dysfunction of PTEN leads to sustained activation of the downstream Akt signaling pathway, which plays a crucial role in the occurrence and development of HCC (Papa and Pandolfi, 2019). Wu et al. (2009) found that the apoptosis-inducing effect of API (20, 40, and 80 µM) in HepG2 cells is associated with the upregulation of PTEN protein expression and the reduction of phosphorylated Akt and phosphorylated Bad protein levels. Subsequently, Yang J. et al. (2018) also confirmed that API (10, 20, and 40 µM) induces apoptosis and autophagy in HCC cells by inhibiting the PI3K/Akt/mTOR signaling pathway. More importantly, inhibiting autophagy significantly enhanced the apoptotic effects of API in HepG2 cells and xenograft models, increasing its anticancer efficacy (Yang J. et al., 2018). This suggests that the combined use of API and autophagy inhibitors may represent a new strategy for treating HCC.

#### 6.1.3 Inhibition of self-renewal of HCC stem cells

Studies have shown that the initiation, progression, local recurrence, distant metastasis, and failure of radiotherapy and chemotherapy in HCC are primarily driven by a small subset of tumor cells with stem cell-like properties, known as cancer stem cells (Lee et al., 2022). Therefore, the cancer stem cell hypothesis has spurred research and development of therapeutic strategies targeting HCC stem cells. Inhibiting the self-renewal ability of HCC stem cells can significantly reduce tumor proliferation and metastasis, thereby improving treatment outcomes (Liu et al., 2020). Fortunately, Jiang et al. (2017) revealed that API (5, 10, and 20 µM) can reduce the tumor sphere formation rate of HCC stem-like cells in a concentration-dependent manner. This inhibition of HCC stem-like cell self-renewal is attributed to the downregulation of CK2α expression by API (Jiang et al., 2017). Similarly, API (10, 20, 40 mg/L) can also reduce the sphere formation rate of MHCC97 cells in a concentration-dependent manner (Jiang et al., 2017). The mechanism is related to the upregulation of Src homology 2 domain-containing protein tyrosine phosphatase 1 (SHP-1) protein expression by API, which in turn reduces the phosphorylation level of STAT3 protein (Jiang et al., 2017). These findings provide strong evidence for API as a potential therapeutic approach targeting the inhibition of self-renewal in HCC stem cells.

#### 6.1.4 Induction of differentiation of HCC cells

HCC cells are typically in an undifferentiated state, characterized by high proliferative capacity and invasiveness, which are major contributors to HCC progression and treatment resistance (Vogel et al., 2022). By inducing differentiation in these cells, their proliferative and invasive capacities can be weakened, and their sensitivity to conventional therapies increased, thereby inhibiting tumor growth and spread (Marquardt et al., 2015). Wen et al. (2007) demonstrated that API (10 µM) can induce differentiation of HepG2 cells from a tumorigenic to a more mature state, with notable changes in morphology and cytoskeletal structure, as well as a significant reduction in γ-GT activity and AFP secretion. It is worth noting that γ-GT is a marker enzyme for HCC, while AFP is a marker of HCC cell differentiation. Similarly, API (5 μm/L) can also induce differentiation in SMMC-7721 cells (Wang, 2018). Additionally, API can enhance the process of differentiation induced by the microenvironment of mouse embryonic liver cells at specific stages, with the synergistic effect peaking at 48 h (Wang, 2018). In summary, these studies suggest that API could serve as a lead compound with differentiation-inducing properties, and its chemical structure may be strategically modified to develop effective differentiation-inducing agents for HCC treatment.

#### 6.1.5 Inhibition of angiogenesis of HCC cells

HCC is a highly vascularized tumor, with its growth and metastasis relying heavily on the process of angiogenesis, or the formation of new blood vessels (Morse et al., 2019). Inhibiting angiogenesis effectively cuts off the tumor’s nutrient supply, restricting its growth and spread, thereby improving patient survival and quality of life (Hu et al., 2022). Typically, the inhibition of angiogenesis is achieved by targeting key molecules such as VEGF and its receptors (Ghalehbandi et al., 2023). Interestingly, Kim et al. (2011) demonstrated that API (40, 80, and 120 µM) induces apoptosis in HCC cells and inhibits cell migration by reducing the expression of stromal proteins and type I collagen. More importantly, API exhibited significant anti-angiogenic effects (Kim et al., 2011). Further studies revealed that the anti-angiogenic activity of API is primarily dependent on the reduction of cell migration activity and the downregulation of VEGF and MMP8 release (Kim et al., 2011). In summary, stromal proteins may play a crucial role in API-induced apoptosis in HCC cells by mediating anti-angiogenic and anti-migratory activities.

Recent studies have indicated that extracellular vesicles (EVs) play a pivotal role in mediating intercellular communication within the tumor microenvironment, greatly influencing the progression of various tumors, including HCC (Kalluri and McAndrews, 2023). Therefore, the comprehensive inhibition of tumor-derived EVs’ production and release could emerge as a more effective strategy to halt tumor progression. Notably, Zhang et al. (2024) revealed that API can inhibit tumor angiogenesis by targeting ARHGEF1, thereby reducing the release of microvesicles and attenuating microvesicle-mediated angiogenesis, ultimately preventing the progression of HCC. This finding not only provides new insights into the anti-tumor mechanisms of API, but also opens up new avenues for curbing HCC progression by targeting and inhibiting the production and function of EVs.

#### 6.1.6 Inhibition of invasion and migration of HCC cells

In anti-HCC therapy, inhibiting the invasion and migration of HCC cells is one of the key strategies (Clark and Vignjevic, 2015). By interfering with the motility and invasive properties of HCC cells, it is possible to effectively block their spread and metastasis. This not only helps in controlling the local spread of HCC but also prevents its metastasis to other organs, thereby improving the quality of life of patients. Qin et al. (2016) demonstrated that API (10, 20 µM) significantly inhibited the proliferation, migration, and invasion of PLC and Bel-7402 cells. Specifically, API reversed the increased levels of epithelial-mesenchymal transition (EMT) markers, enhanced cell adhesion, and regulated actin polymerization and cell migration (Qin et al., 2016). These effects are likely attributable to its inhibition of the NF-κB/Snail signaling pathway (Qin et al., 2016).

Additionally, API (5, 10, and 20 mg/L) was shown to inhibit the invasion of Huh-7 cells in a dose-dependent manner (Wang, 2020). Further transcriptomic sequencing results suggest that API may suppress cell invasion by modulating the differential expression of miRNAs (Wang, 2020). Zhou (Zhou, 2021) further elucidated the mechanisms by which API regulates HCC cell migration and invasion. The data indicate that API inhibits HCC cell migration and invasion, at least in part, through the downregulation of YAP, a downstream effector molecule of the Hippo pathway (Wang, 2020). These findings suggest that API may be an effective alternative therapy for treating refractory cancers and holds promise as an anti-metastatic candidate drug. The hepatoprotective effects and mechanisms of API in liver cancer (monotherapy) are presented in Table 5.

**TABLE 5:** The functions and molecular mechanisms of API in liver cancer (monotherapy).

Models	Types	Routes	Dosage of administration	Molecular mechanisms	Years	References
HepG2 cells	*In vitro*	N/A	0.625–10 µM API for 24–72 h	Inhibition of cell proliferation and glucose uptake in HCC cells	2015	Chen et al. (2015)
BALB/c nude mice + HepG2 cells	*In vivo*	i.p.	0.5 mg/kg API for 30 days (Every other day)	Inhibition of tumor growth exerts anti-tumor effects	2017	Li et al. (2017)
HepG2 cells	*In vitro*	N/A	0.625–10 µM API for 24–72 h	Inhibited the growth and proliferation of liver cancer cells	2018	Bhattacharya et al. (2018)
Huh-7 cells.	*In vitro*	N/A	0.625–10 µM API for 24–72 h			
Sprague Dawley male rats + HepG2 cells	*In vivo*	i.v.	20 mg/kg API for 12 weeks			
LO2 cells	*In vitro*	N/A	10, 20, 30, 40 µM API for 24–72 h	Through H19-mediated Wnt/β-catenin signaling regulatory axis	2021	Pan et al. (2021)
SMMC-7721 cells	*In vitro*	N/A	10, 20, 30, 40 µM API for 24–72 h			
HepG2 cells	*In vitro*	N/A	10, 20, 30, 40 µM API for 24–72 h			
Male nude mice + HepG2 cells	*In vivo*	i.p.	50 mg/kg API for 4 weeks (Every other day)			
Male nude mice + HepG2 cells	*In vivo*	i.g.	50 mg/kg API for 4 weeks (Every other day)			
MHCC97H cells	*In vitro*	N/A	50, 100 µM API for 24 h	Regulation of lipid metabolism in HCC cells by targeting KDM1A	2020	Zhang (2020)
HepG2 cells	*In vitro*	N/A	50, 100 µM API for 24 h			
HepG2 cells	*In vitro*	N/A	10, 20, 50 µM API for 24 h	Inhibition of cell growth through MED28-mediated mTOR signaling	2024	Chou et al. (2024)
Huh 7 cells	*In vitro*	N/A	10, 20, 50 µM API for 24 h			
HepG2 cells	*In vitro*	N/A	4, 8, 16 mg/mL API for 48 h	Through the p53-dependent pathway and the induction of p21 expression	2006	Chiang et al. (2006)
Hep3B cells	*In vitro*	N/A	4, 8, 16 mg/mL API for 48 h			
PLC/PRF/5 cells	*In vitro*	N/A	4, 8, 16 mg/mL API for 48 h			
HepG2 cells	*In vitro*	N/A	25, 50 µM API for 12–48 h	TNF-α and IFN-γ processes mediate the plausible mechanism of apoptosis	2006	Khan and Sultana (2006)
HepG2 cells	*In vitro*	N/A	20, 40, 80 µM API for 48 h	Up-regulating PTEN and reducing phosphorylated Akt and Bad protein level	2009	Wu et al. (2009)
HCC hepatocytes	*In vitro*	N/A	10, 20, 40 µM API for 5–60 min	Inhibition of tumor growth by direct targeting of mitochondria	2016	Seydi et al. (2016)
HepG2 cells	*In vitro*	N/A	10, 20, 40 µM API for 12 h	Inhibits cell proliferation and induces autophagy via suppressing the PI3K/Akt/mTOR pathway	2018	Yang J. et al. (2018)
HepG2 cells	*In vitro*	N/A	130.2 µM API for 24 h	Induction of apoptosis	2021	Ganguly et al. (2021)
SMMC-7721 cells	*In vitro*	N/A	5, 10, 20 µM API for 6 days	Downregulation of CK2α expression	2017	Jiang et al. (2017)
MHCC97H cells	*In vitro*	N/A	10, 20, 40 mg/L API for 48 h	Reduced phosphorylation level of STAT3 protein by upregulation of SHP-1 expression	2018	Jiang et al. (2017)
HepG2 cells	*In vitro*	N/A	10 µM API for 48 h	Promoting cell differentiation in HCC	2007	Wen et al. (2007)
SMMC-7721 cells	*In vitro*	N/A	2.5, 5, 7.5, 10, 12.5 μm/L API for 24–72 h	Induction of differentiation	2018	Wang (2018)
Huh7 cells	*In vitro*	N/A	40, 80, 120 µM API for 24 h	Downregulation of vimentin, type I collagen, VEGF and MMP8	2011	Kim et al. (2011)
DEN and CCl_4_-induce primary liver cancer in C57BL/6 mice	*In vivo*	i.p.	25 mg/kg API for 30 days (Every other day)	By hindering microvesicle biogenesis via ARHGEF1	2024	Zhang et al. (2024)
HCC-LM3 cells	*In vitro*	N/A	25 µM API for 48 h			
PLC/PRF/5 cells	*In vitro*	N/A	25 µM API for 48 h			
MHCC-97H cells	*In vitro*	N/A	25 µM API for 48 h			
Huh7 cells	*In vitro*	N/A	25 µM API for 48 h			
HepG2 cells	*In vitro*	N/A	25 µM API for 48 h			
Hep3B cells	*In vitro*	N/A	25 µM API for 48 h			
MDA-MB-231 cells	*In vitro*	N/A	25 µM API for 48 h			
Bel-7402 cells	*In vitro*	N/A	10, 20 µM API for 24 h	Inhibiting the NF-κB/Snail pathway	2016	Qin et al. (2016)
PLC/PRF/5 cells	*In vitro*	N/A	10, 20 µM API for 24 h			
BALB/c nu/nu mice + tumor cell	*In vivo*	i.p.	200, 300 mg/kg API for 7 weeks			
Male nude mice + Huh7 cells	*In vivo*	i.p.	50 μg/d API for 20 days	Regulation of the proteoglycans in cancer and microRNAs in cancer pathways	2020	Wang (2020)
Huh7 cells	*In vitro*	N/A	5, 10, 20 mg/L API for 48 h			
SMMC-7721 cells	*In vitro*	N/A	20, 30, 60 µM API for 24–72 h	Regulation of the expression of key EMT and autophagy-related genes by downregulation of the Hippo signaling pathway effector molecule YAP	2021	Zhou (2021)
SK-HEP1 cells	*In vitro*	N/A	10, 20, 40 µM API for 24–72 h			

### 6.2 Combination therapy

#### 6.2.1 With sorafenib

Sorafenib (C_21_H_16_ClF_3_N_4_O_3_, SOR) is a multi-targeted oral anticancer drug widely used in the treatment of advanced HCC, advanced renal cell carcinoma, and differentiated thyroid carcinoma (Escudier et al., 2019). It exerts its antitumor effects by inhibiting various intracellular and extracellular kinases, thereby interfering with tumor cell proliferation and angiogenesis, which inhibits tumor growth and metastasis (Xia et al., 2020). Despite its remarkable efficacy in cancer treatment, SOR is associated with adverse effects that cannot be ignored, including rash, hand-foot syndrome, hypertension, and diarrhea (Gauthier and Ho, 2013; Keating, 2017). Consequently, researchers are exploring combination therapy strategies to overcome SOR resistance and reduce its adverse effects.

Interestingly, Şirin et al. (2020) were the first to investigate the effect of combining SOR with API on HepG2 cells. The data demonstrated that combination therapy significantly decreased cell viability, increased apoptosis, and inhibited cell migration and invasion (Şirin et al., 2020). These findings suggest that API accelerates and enhances the anticancer effects of SOR while exhibiting lower toxicity to healthy cells, potentially helping to overcome SOR resistance (Şirin et al., 2020). However, further research is needed to determine the optimal dosage and method of this combination therapy. Similarly, Singh et al. (2024) also demonstrated that the combination of SOR and API was more effective than SOR alone, exhibiting stronger cytotoxicity, apoptosis induction, tumor cell cycle arrest, and tumor growth inhibition. Overall, API emerges as a promising adjuvant, and further studies will help to deepen our understanding of its mechanism of action.

#### 6.2.2 With doxorubicin

Doxorubicin (C_27_H_29_NO_11_, DOX) is an anthracycline antibiotic widely used to treat various cancers, including breast cancer, ovarian cancer, bladder cancer, lung cancer, and lymphomas (Kciuk et al., 2023). Its mechanism of action involves intercalating into the DNA double helix, interfering with DNA and RNA synthesis, thereby inhibiting cell proliferation and inducing apoptosis (van der Zanden et al., 2021). Despite its significant antitumor effects in clinical applications, DOX use is associated with severe side effects, particularly cardiotoxicity (Sheibani et al., 2022). Therefore, researchers have been striving to enhance the therapeutic efficacy of DOX while reducing its side effects.

Studies have shown that API can enhance the sensitivity of DOX-resistant BEL-7402 (BEL-7402/ADM) cells to DOX (Gao et al., 2013). Specifically, in a BEL-7402 xenograft model, the combination of API and DOX significantly inhibited tumor growth, reduced cell proliferation, and induced apoptosis compared to DOX alone (Gao et al., 2013). This beneficial effect may be attributed to the inhibition of the PI3K/Akt/Nrf2 signaling pathway, thereby reversing the resistant phenotype (Gao et al., 2013). Four years later, the research team further discovered that API could sensitize BEL-7402/ADM cells to DOX by modulating the miR-520b/ATG7-related autophagy pathway in HCC (Gao et al., 2018). These findings not only help to understand the potential molecular mechanisms of chemotherapy resistance but also provide new therapeutic strategies to overcome drug resistance in liver cancer.

#### 6.2.3 With paclitaxel

Paclitaxel (C_47_H_51_NO_14_, PTX) is a widely used clinical anticancer drug primarily used to treat various solid tumors, including breast cancer, ovarian cancer, non-small cell lung cancer, and pancreatic cancer (Liu et al., 2023). Its primary mechanism of action involves promoting the polymerization of tubulin, stabilizing microtubule structures, thereby preventing the normal depolymerization of microtubules during cell division, leading to cell cycle arrest at mitosis, and ultimately inducing apoptosis (Zhu and Chen, 2019). However, PTX resistance remains a significant challenge in treatment. Interestingly, Li et al. (2020) revealed that API significantly reduced hypoxia-induced resistance to PTX in HepG2 cells and tumor-bearing mouse models. Mechanistically, API enhanced the anticancer activity of PTX in hypoxic tumors by inhibiting the Akt/p-Akt pathway and HSP90 expression, thereby reducing HIF-1α expression (Li et al., 2020). These results not only confirm the synergistic effect of PTX and API but also highlight the potential of using API in combination with other anticancer drugs as adjuvant therapy.

#### 6.2.4 With cisplatin

Cisplatin (PtCl_2_(NH_3_)_2_, CDDP) is a platinum-based anticancer drug widely used in clinical practice to treat various solid tumors, including testicular cancer, ovarian cancer, bladder cancer, head and neck cancers, and lung cancer (Ghosh, 2019). The mechanism of action is to induce apoptosis and programmed cell death through the formation of CDDP-DNA adducts, leading to intra- and interstrand cross-linking of DNA and interference with DNA replication and transcription processes (Rocha et al., 2018). Despite its broad-spectrum antitumor activity, CDDP use is also associated with side effects, including nephrotoxicity, ototoxicity, and neurotoxicity (McSweeney et al., 2021). Moreover, CDDP resistance is an issue that needs to be addressed. Studies have shown that API can enhance the chemotherapeutic effects of CDDP in liver cancer cells, including HepG2, Hep3B, and Huh7 cells (Papachristou et al., 2021). Specifically, API enhanced the genotoxicity, cytotoxicity, anti-invasive, and anti-migratory effects of CDDP in HCC cell lines (Papachristou et al., 2021). These findings provide direction for future exploration of the precise mechanisms of action when using CDDP and API in combination.

#### 6.2.5 With 5-fluorouracil

5-Fluorouracil (C_4_H_3_FN_2_O_2_, 5-FU) is a chemotherapeutic drug widely used in the treatment of various malignancies, including colorectal cancer, gastric cancer, pancreatic cancer, and breast cancer (Hu et al., 2023). Its mechanism of action involves inhibiting the key enzyme in the pyrimidine metabolic pathway—thymidylate synthase—thereby interfering with DNA and RNA synthesis, leading to the inhibition of cancer cell proliferation and growth (Sethy and Kundu, 2021). However, 5-FU is also associated with certain side effects, including bone marrow suppression, gastrointestinal reactions, and mucositis (Wei et al., 2018). Therefore, it is often used in combination with other chemotherapeutic drugs (such as oxaliplatin, irinotecan) or targeted therapies (such as bevacizumab, ramucirumab) to enhance therapeutic efficacy and reduce resistance.

Research indicates that the combination of API with 5-FU may be an effective chemotherapy strategy for HCC (Hu et al., 2015). Specifically, the combination treatment significantly increased reactive ROS levels in liver cancer cells, leading to a decrease in mitochondrial membrane potential and activation of the mitochondrial apoptosis pathway (Hu et al., 2015). Mechanistically, API may enhance the chemosensitivity of liver cancer cells to 5-FU by activating the intrinsic apoptosis pathway mediated by mitochondrial membrane potential (Hu et al., 2015). This discovery provides a new avenue for optimizing chemotherapy regimens for HCC and overcoming chemotherapy resistance.

#### 6.2.6 With chrysin

**FIGURE 4:**
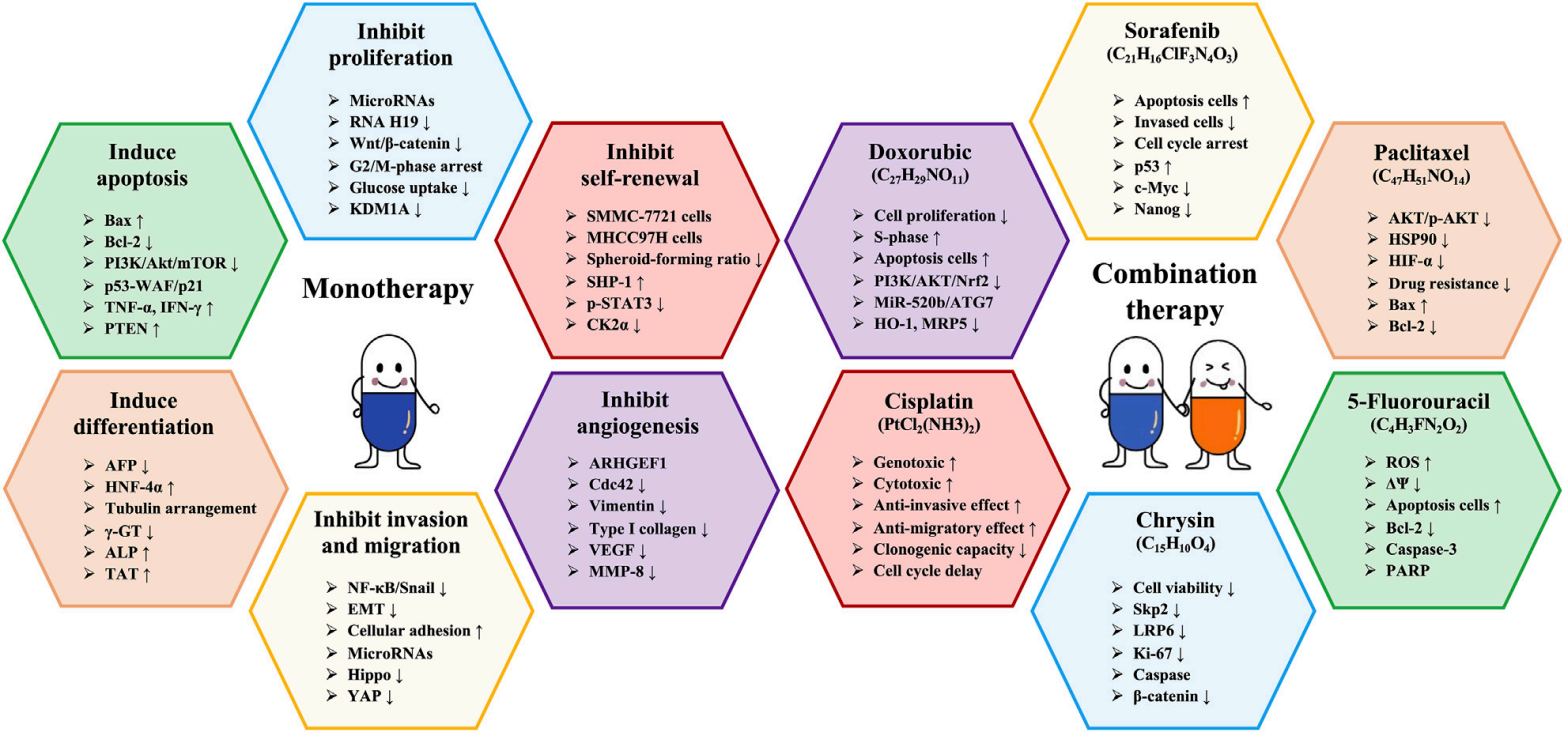
Schematic diagram of API improving liver cancer.

Chrysin (C_15_H_10_O_4_, CHR) is a naturally occurring flavonoid compound found in propolis, bee pollen, and various plants, garnering attention for its multiple biological activities, including antioxidant, anti-inflammatory, anticancer, and neuroprotective effects (Mani and Natesan, 2018). Of particular importance is CHR’s anticancer activity, which involves mechanisms such as inducing apoptosis, inhibiting proliferation, and preventing invasion and metastasis of cancer cells (Salari et al., 2022). Interestingly, Huang et al. (2016) demonstrated that the combination of API and CHR significantly reduced the viability of HepG2 and MDA-MB-231 cells, induced apoptosis, and reduced the migratory capacity of MDA-MB-231 cells. Moreover, combination therapy effectively inhibited the growth of MDA-MB-231 xenograft tumors (Huang et al., 2016). These results indicate that the combination of CHR and API has potential anticancer effects, providing a solid foundation for the research of new drug targets. The hepatoprotective effects and mechanisms of API in liver cancer (combination therapy) are presented in Table 6. The schematic diagram of API improvement of liver cancer is shown in Figure 4.

**TABLE 6:** The functions and molecular mechanisms of API in liver cancer (combination therapy).

Co-administered drugs	Combined drug dosage	Models	Types	Routes	Dosage of administration	Molecular mechanisms	Years	References
Sorafenib (C_21_H_16_ClF_3_N_4_O_3_)	100 µM	HepG2 cells	*In vitro*	N/A	10–100 µM API for 24–72 h	Through effects on cell migration, invasion, apoptosis and gene expression	2020	Şirin et al. (2020)
Sorafenib (C_21_H_16_ClF_3_N_4_O_3_)	2 µM	HepG2 cells	*In vitro*	N/A	48 μM API for 48 h	Intensifying its cytotoxic effects, triggering apoptosis, promoting cell cycle arrest, and impeding tumor growth in animal models	2024	Singh et al. (2024)
	2 µM	Huh7 cells	*In vitro*	N/A	48 μM API for 48 h			
	10 mg/kg	DEN-induced hepatocarcinogenesis in Swiss Albino male mice	*In vivo*	i.p.	50 mg/kg API for 17 weeks (Every other day)			
Doxorubicin (C_27_H_29_NO_11_)	8 μM	BEL-7402 cells	*In vitro*	N/A	10, 20 µM API for 24 h	Inhibition of PI3K/Akt/Nrf2 pathway	2013	Gao et al. (2013)
	3 mg/kg	BALB/c nude male mice + BEL-7402 cells	*In vivo*	i.p.	50 mg/kg API for 21 days (Every 3 days)			
Doxorubicin (C_27_H_29_NO_11_)	2 μM	BEL-7402 cells	*In vitro*	N/A	25, 50 µM API for 12–48 h	Regulation of miR-520b/ATG7 axis	2018	Gao et al. (2018)
	3 mg/kg	Male nude mice + HepG2 cells	*In vivo*	i.p.	50 mg/kg API for 21 days (Every 3 days)			
Paclitaxel (C_47_H_51_NO_14_)	100 nM	HepG2 cells + BALB/c nude mice	*In vitro*	N/A	10, 40, 100 µM API for 24 h	Inhibiting the Akt/p-Akt pathway and the expression of HSP90	2020	Li et al. (2020)
	3.5 mg/kg	Balb/c male nude mice + HepG2 cells	*In vivo*	i.v.	1 mg/kg API for 20 days (Every other day)			
Cisplatin (PtCl_2_(NH_3_)_2_)	0.025, 0.25, 2.5 μg/mL	HepG2 cells	*In vitro*	N/A	10, 20 µM API for 24 h	Enhanced CDDP’s genotoxic, cytotoxic, anti-invasive, and anti-migratory effect on liver cancer cell lines	2021	Papachristou et al. (2021)
	0.025, 0.25, 2.5 μg/mL	Hep3B cells	*In vitro*	N/A	10, 20 µM API for 24 h			
	0.025, 0.25, 2.5 μg/mL	Huh7 cells	*In vitro*	N/A	10, 20 µM API for 24 h			
5-Fluorouracil (C_4_H_3_FN_2_O_2_)	25 mg/mL	Male nude mice + SK-Hep-1 cells	*In vivo*	i.p.	100 μL API for 3 weeks (5 days a week)	Inhibition of drug resistance mediated by ROS and simultaneous activation of mitochondrial apoptosis pathway	2015	Hu et al. (2015)
	100 μg/mL	SK-Hep-1 cells	*In vitro*	N/A	4 µM API for 48 h			
	100 μg/mL	BEL-7402 cells	*In vitro*	N/A	4 µM API for 48 h			
Chrysin (C_15_H_10_O_4_)	10 μM	HepG2 cells	*In vitro*	N/A	10 μM API for 72–96 h	Decreased proliferation and cell viability of HepG2 and MDA-MB-231, and induced apoptosis	2016	Huang et al. (2016)
	10 μM	AU565 cells	*In vitro*	N/A	10 μM API for 72–96 h			
	10 μM	MDA-MB-231 cells	*In vitro*	N/A	10 μM API for 72–96 h			
	10 mg/kg	BALB/c female nude mice + MDA-MB-231 cells	*In vivo*	i.p.	10 mg/kg API for 28 days			

## 7 Toxicity of API

Most studies have reported that API exhibits no significant toxicity to cells or experimental animals. For instance, Seydi et al. (2016) demonstrated that API exhibited no cytotoxicity to normal liver cells at concentrations ranging from 0 to 50 μM and showed only minimal cytotoxicity at 100 μM. Similarly, Vrhovac Madunić et al. (2018) found that API exhibited no apparent toxicity to human peripheral blood lymphocytes (HPBLs) at concentrations ranging from 0 to 100 μM, and regardless of concentration and exposure time, did not significantly increase the number of DNA strand breaks. It is noteworthy that Wang (Wang, 2022) explored the impact of API-casein nanoparticles on organ coefficients and liver toxicity in rats. The results showed that oral administration of API-casein nanoparticles (equivalent to API 100–1,000 mg/kg) for 2 weeks had no significant effect on the organ coefficients of the heart, liver, spleen, lungs, and kidneys, nor on the hepatic histological structure in rats, indicating good biosafety (Wang, 2022). Overall, these studies suggest that API exhibits extremely low toxicity and has a high degree of clinical safety, with broad prospects for development and application. However, systematic toxicological studies are still needed in the future to comprehensively assess the toxicity and mechanisms of API.

## 8 Pharmacokinetic properties of API

It is surprising that approximately 40% of failures in drug development can be attributed to inappropriate pharmacokinetic properties (Walker, 2004). Therefore, determining the pharmacokinetic characteristics of a drug in the early stages of development is crucial. Pharmacokinetic analysis allows for the prediction of individual differences in drug behavior, optimization of drug efficacy and safety, and reduction of adverse reactions (van den Anker et al., 2018). Additionally, pharmacokinetic data provide a scientific basis for drug interactions and dosing in special populations, thereby improving the effectiveness and safety of drug therapy (Koziolek et al., 2019). Currently, the pharmacokinetics of API are mainly studied using high-performance liquid chromatography-tandem mass spectrometry (HPLC-MS/MS) and ultra-performance liquid chromatography-tandem mass spectrometry (UPLC-MS/MS) to analyze plasma samples from rats and mice. Comparative pharmacokinetic parameters of API from seven studies are summarized in Table 7.

**TABLE 7:** Pharmacokinetic properties of API.

Route of administration	Species	Dose (mg/kg, equivalent to API)	Pharmacokinetic parameters								References
			T_max_ (h)	C_max_ (a:μg/mL; b: Ng/mL; c: μM; d: μg/L)	AUC_(0-t)_ (a: ng·h/mL; b: μg·h/mL; c: μM·h; d: μg·h/L)	AUC_(0-∞)_ (a: μg·h/mL; b:μM·h; c: μg·h/L; d: ng·h/mL)	T_1/2_ (h)	CL (a: mL/h/kg; b: L/h/kg)	MRT_(0-t)_ (h)	Vz (a: mL; b: L)	
Oral	Rats (Wistar, male)	10 (Coarse powder)	3.60 ± 1.67	21.38 ± 14.35 (a)	146.54 ± 139.62 (a)	N/A	N/A	N/A	N/A	N/A	Elzayat et al. (2019)
Oral	Rats (Wistar, male)	10 (Commercial product)	4.00 ± 1.63	17.47 ± 2.96 (a)	141.97 ± 67.24 (a)	N/A	N/A	N/A	N/A	N/A	
Oral	Rats (Wistar, male)	10 (Microwave treated)	2.80 ± 1.30	60.54 ± 24.70 (a)	453.20 ± 328.78 (a)	N/A	N/A	N/A	N/A	N/A	
Oral	Rats (Wistar, male)	10 (Kneaded)	3.20 ± 1.78	11.76 ± 4.41 (a)	83.35 ± 19.41 (a)	N/A	N/A	N/A	N/A	N/A	
Oral	Rats (Wistar, male)	10 (Melted)	2.80 ± 1.78	31.78 ± 21.21 (a)	187.36 ± 92.40 (a)	N/A	N/A	N/A	N/A	N/A	
Oral	Rats (Wistar, male)	10 (Spray dried)	1.20 ± 0.44	92.39 ± 53.52 (a)	344.40 ± 134.50 (a)	N/A	N/A	N/A	N/A	N/A	
Oral	Rats (Wistar, male)	10 (SNEDDS)	4.20 ± 2.04	43.84 ± 20.12 (a)	280.37 ± 58.62 (a)	N/A	N/A	N/A	N/A	N/A	
Oral	Rats (Wistar, female)	10	24.00 ± 0.00	6.91 ± 0.54 (a)	N/A	1,038.00 ± 64.40 (a)	91.80 ± 5.60	1.95 ± 0.11 (a)	N/A	259.10 ± 25.80 (a)	Gradolatto et al. (2005)
Oral	Rats (SD, male)	60	2.50 ± 0.33	1.33 ± 0.24 (a)	11.76 ± 1.52 (b)	12.02 ± 2.61 (a)	4.20 ± 0.29	N/A	N/A	N/A	Ding et al. (2014)
Oral	Rats (SD, male)	60 (API-SD)	2.00 ± 0.28	3.26 ± 0.33 (a)	21.48 ± 2.83 (b)	26.53 ± 53.84 (a)	3.37 ± 0.46	N/A	N/A	N/A	
Oral	Rats (SD, male)	60 (API-PM)	2.50 ± 0.36	1.43 ± 0.18 (a)	13.55 ± 1.44 (b)	13.90 ± 3.19 (a)	4.34 ± 0.32	N/A	N/A	N/A	
Oral	Rats (SD, male)	13.51	0.50 ± 0.01	42.00 ± 2.00 (b)	659.00 ± 25.00 (a)	N/A	2.11 ± 0.03	N/A	N/A	N/A	Teng et al. (2012)
i.v.	Rats (SD, male)	20	N/A	10,933.88 ± 1730.11 (d)	3,211.54 ± 554.88 (d)	3,312.10 ± 473.30 (c)	1.75 ± 1.18	6.12 ± 0.79 (b)	0.65 ± 0.50	15.75 ± 11.73 (b)	Wan et al. (2007)
i.v.	Rats (Wistar, male)	2.49	N/A	0.08 ± 0.00 (a)	1.41 ± 0.05 (b)	1.87 ± 0.03 (a)	26.60 ± 1.20	1.33 ± 0.03 (b)	33.51 ± 2.11	N/A	Ganguly et al. (2021)
i.v.	Rats (Wistar, male)	2.49 (API-NPs)	N/A	0.06 ± 0.00 (a)	2.67 ± 0.04 (b)	5.22 ± 0.03 (a)	70.79 ± 1.11	0.48 ± 0.02 (b)	100.88 ± 2.23	N/A	
i.v.	Rats (Wistar, male)	2.49 (API-GAL-NPs)	N/A	0.06 ± 0.00 (a)	2.99 ± 0.04 (b)	6.98 ± 0.04 (a)	92.28 ± 1.23	0.36 ± 0.01 (b)	128.75 ± 2.66	N/A	
i.v.	Rats (SD, male)	2	N/A	15.50 ± 0.16 (b)	125.80 ± 52.00 (a)	130.61 ± 53.40 (d)	5.25 ± 0.25	15.90 ± 0.55 (b)	12.81 ± 1.58	N/A	Dhara et al. (2023)
i.v.	Rats (SD, male)	2 (NLCs)	N/A	17.20 ± 1.13 (b)	496.40 ± 62.90 (a)	539.64 ± 79.34 (d)	13.51 ± 0.20	4.03 ± 0.63 (b)	30.83 ± 1.09	N/A	
i.v.	Rats (SD, male)	2 (PEG-NLCs)	N/A	18.10 ± 0.55 (b)	826.40 ± 53.00 (a)	1,072.47 ± 171.21 (d)	39.02 ± 0.20	2.42 ± 0.66 (b)	36.94 ± 0.41	N/A	
i.v.	Rats (SD, male)	2 (Apt-NLCs)	N/A	19.20 ± 0.51 (b)	986.20 ± 47.00 (a)	1,446.21 ± 182.65 (d)	43.02 ± 0.25	2.03 ± 0.52 (b)	38.91 ± 0.21	N/A	

Note: T_max_, time to maximum plasma concentration; C_max_, maximum plasma concentration; AUC_(0–t)_, area under the concentration-time curve from 0 to t h; AUC_(0-∞)_, area under the concentration-time curve from 0 h to infinity; T_1/2_, elimination half-life; CL, clearance; MRT_(0–t)_, mean residence time from 0 to t h; Oral, oral administration; i.v., intravenous administration; Vz, clearance, vertical distribution phase; SNEDDS, self-nanoemulsifying drug delivery system; API-SD, API, solid dispersion; API-PM, API physical mixture; API-NPs, API nanoparticles; API-GAL-NPs, API, galactose nanoparticles; NLCs, API-encapsulated plain nanoliposomes; PEG-NLCs, PEGylated NLCs; Apt-NLCs, aptamer functionalized PEGylated NLCs; N/A, not applicable.

Ganguly et al. (2021) indicates that API exhibits slow distribution and elimination rates in rats. Following intravenous administration (2.49 mg/kg), the T_1/2_, C_max_, AUC_(0-t)_, and CL of API were 26.60 ± 1.20 h, 0.08 ± 0.00 μg/mL, 1.41 ± 0.05 μg/mL·h, and 1.33 ± 0.03 L/h/kg, respectively (Ganguly et al., 2021). Additionally, another study reported that the terminal phase distribution volume (Vd) of API after intravenous administration (20 mg/kg) was 15.75 ± 11.73 L/kg, exceeding the total body water volume in rats, suggesting that API was well-distributed into tissues (Wan et al., 2007). Meanwhile, the total body clearance (CL) of API following intravenous administration (20 mg/kg) was 6.12 ± 0.79 L/h/kg, significantly higher than the hepatic blood flow rate in rats, indicating that API is cleared not only through hepatic metabolism but also via renal or other excretory pathways (Wan et al., 2007).

Currently, the elimination pathways of API have not been systematically explored. Existing studies suggest that the half-life (T_1/2_) of API remains uncertain. For oral doses ranging from 10 to 60 mg/kg, the T_1/2_ ranges from 2.11 to 91.8 h (Gradolatto et al., 2005; Teng et al., 2012; Ding et al., 2014). For injection doses of 2–20 mg/kg, the T_1/2_ ranges from 1.75 to 26.6 h (Wan et al., 2007; Ganguly et al., 2021; Dhara et al., 2023). Interestingly, another study also pointed out that API has a slow absorption and metabolism process in rats, primarily excreted through urine (Gradolatto et al., 2005). Importantly, a gender difference in the metabolic rate of API was observed, with female rats exhibiting a slightly higher metabolic rate than male rats, especially in the formation of glucuronide conjugates (Gradolatto et al., 2005). Therefore, future research should delve into the metabolic differences of API across different genders and physiological states to formulate personalized dosing regimens in clinical applications.

Due to the limitations in the absorption and bioavailability of API, some researchers have focused on enhancing its oral bioavailability through experimental approaches. For instance, Ding et al. (2014) developed a novel nanocarbon drug carrier, which significantly improved the dissolution rate and oral bioavailability of API. Specifically, the C_max_ and AUC_(0-t)_ values of API-SD were 2.45-fold and 1.83-fold higher than those of API, respectively, with a relative oral bioavailability increase of 183% (Ding et al., 2014). Similarly, Ganguly et al. (2021) developed galactose-modified PLGA nanoparticles (API-GAL-NPs) loaded with API for active liver-targeted treatment of HCC. Notably, the AUC_(0-t)_, T_1/2_, and MRT_(0-t)_ values of API-GAL-NPs were 2.12-fold, 3.47-fold, and 3.84-fold higher than those of API, respectively, indicating that API-GAL-NPs could maintain stable and significantly higher levels of API in the blood, offering superior anticancer effects compared to API alone (Ganguly et al., 2021). In conclusion, future studies should further explore the pharmacokinetic properties of API in various animal models and evaluate its long-term safety and potential toxicity.

## 9 New formulations of API

Despite the significant bioactivity and numerous health benefits of API, its clinical application is hindered by factors such as low bioavailability, poor water solubility, and drug interactions. Therefore, improving drug design and formulation technologies to overcome these limitations is of paramount importance. Interestingly, the development of novel drug delivery systems offers an effective approach to address these challenges associated with API.

### 9.1 Nanoparticles

#### 9.1.1 Polymer nanoparticles

Polymeric nanoparticles (PNPs) are composed of natural or synthetic polymers and are widely used in drug delivery, gene therapy, and tissue engineering (Zielińska et al., 2020). Notably, polymeric nanoparticles can achieve controlled drug release *in vivo* by modulating parameters such as the composition, size, and surface charge of the nanoparticles (Elmowafy et al., 2023). Remarkably, polymeric micelles loaded with API, prepared by Zhai et al. (2013), effectively improved the solubility of API in water. *In vitro* drug release studies showed that nearly 84% of API was released from the micelles within 36 h, exhibiting significant sustained-release characteristics (Zhai et al., 2013). Further experiments demonstrated that polymeric micelles loaded with API exhibited significantly higher toxicity to HepG2 and MCF-7 cells compared to free API (Zhai et al., 2013). Subsequently, Das et al. (2013) developed API-loaded PLGA nanoparticles and found that they enhanced the anticancer effects in mice with skin tumors and mitochondrial dysfunction induced by Benzo [a]pyrene and UV-B radiation.

In recent years, numerous studies have demonstrated that API-loaded nanoparticles have significant anti-HCC effects. For instance, Bhattacharya et al. (2018) showed that API-loaded nanoparticles significantly increased the concentration of API in the blood and liver, substantially controlling the progression of HCC. This beneficial effect may be attributed to the enhanced permeability and retention (EPR) effect of the API-loaded nanoparticles in solid tumors (Bhattacharya et al., 2018). Additionally, Ganguly et al. (2021) confirmed that API-loaded galactose-modified PLGA nanoparticles (API-GAL-NPs) exhibited more pronounced therapeutic effects in rats with diethylnitrosamine (DEN)-induced liver cancer. This superior efficacy is likely achieved through the active targeting and improved internalization of API by API-GAL-NPs in liver cancer cells (Ganguly et al., 2021).

Interestingly, Dhara et al. (2023) developed a tumor-responsive phosphorothioated and amino-modified aptamer (AS1411)-conjugated stealth nanoliposome for encapsulating API, successfully achieving targeted drug distribution of API in the tumor. More importantly, this aptamer-modified nanoliposome (Apt-NLCs) significantly reduced the incidence of tumors and tumor-associated hepatic degenerative lesions, further validating its potential in the treatment of HCC (Dhara et al., 2023). These research findings indicate that the application of nanotechnology has significantly improved the drug delivery and therapeutic efficacy of API, providing a solid foundation for its clinical application.

#### 9.1.2 Metal nanoparticles

Metal nanoparticles (MNPs) have shown great potential in areas such as biosensing, drug delivery, and cancer therapy due to their high surface area and unique optical properties (Sánchez-López et al., 2020). Among them, silver nanoparticles have gained attention for their powerful antibacterial properties, capable of exerting broad-spectrum antibacterial effects by releasing silver ions that disrupt bacterial cell membranes and DNA structures (Durán et al., 2016). One study demonstrated that silver nanoparticles synthesized with API (AP-SNPs) exhibited enhanced anticancer, antibacterial, and antioxidant activities (Zarei et al., 2021). Specifically, AP-SNPs improved liver function by modulating liver enzymes, lipid peroxidation, and increasing the expression of antioxidant enzymes (Zarei et al., 2021). Although AP-SNPs have shown great potential, further research and evaluation are needed to assess their potential biotoxicity and environmental impact to ensure their safe and effective clinical application.

#### 9.1.3 Carbon-based nanoparticles

Carbon-based nanoparticles (CBNPs) hold an important position in nanotechnology and biomedicine, with major types including carbon nanotubes, graphene, fullerenes, and carbon nanopowder (CNP) (Patel et al., 2019). Carbon nanopowder has become a research hotspot as a drug carrier due to its high surface area, chemical stability, and biocompatibility. Its unique surface properties enable it to load various drugs through physical adsorption or chemical bonding, thereby enhancing drug solubility and bioavailability (Johnson et al., 2022). Ding et al. (2014) demonstrated that carbon nanopowder solid dispersions significantly increased the bioavailability of API by up to 183%. This effect was partly attributed to the increased dissolution and absorption rates in rats (Ding et al., 2014).

#### 9.1.4 Other nanoparticles

Li (2008), Wang (2012); Wang (2022), and Huang et al. (2019b), among others, successfully prepared different types of API nanocarriers using various methods such as hot-melt ultrasonic dispersion, phase inversion, acid-base, and physical adsorption. These nanocarriers included API solid lipid nanoparticles, API liposome nanocapsules, API-casein nanoparticles, and API-loaded mesoporous silica nanoparticle solid dispersions (AP-MSN). These novel nanocarriers significantly improved the oral absorption and bioavailability of API, particularly the API-casein nanoparticles, which significantly enhanced the solubility of API in simulated gastric and intestinal fluids by 422.96-fold and 108.20-fold, respectively, compared to pure API (Wang, 2022). As the main natural protein in milk, casein has good biocompatibility and safety. Therefore, using it as a carrier material to construct an oral delivery system for API not only enhances the stability of API but also promotes its absorption and utilization in the body.

### 9.2 Nanosuspensions

Nanosuspensions are stable dispersion systems formed by dispersing drug microparticles or nanoparticles in a liquid medium, significantly improving the solubility, dissolution rate, and bioavailability of poorly soluble drugs (Chavhan, 2024). API nanosuspensions, prepared by Yu (2015) using a micro-precipitation-ultrasonic method, exhibited a drug loading of 44.4% ± 0.06% and achieved a release rate of 80% within 72 h in 20% EtOH/PBS. More importantly, API nanosuspensions increased the relative bioavailability of API by enhancing the C_max_, AUC_(0-t)_, and MRT_(0-t)_ parameters (Yu, 2015). Subsequently, Lv (2016) further demonstrated that API nanosuspensions improved the distribution of API in mice, increasing liver uptake and favoring drug targeting to liver tissue. Moreover, compared to API, the nanosuspensions significantly enhanced the toxicity to HepG2 cells, increased the apoptosis rate of HepG2 cells, strengthened G2/M phase arrest, and improved the inhibition rate of liver tumors in nude mice (Lv, 2016). In summary, API nanosuspensions significantly enhance the solubility and bioavailability of the drug, thereby improving its pharmacological activity, and showcasing broad application prospects and clinical translation value.

### 9.3 Others

Phospholipid phytosomes are complexes formed by combining active plant ingredients with phospholipids, widely used in drug delivery and nutritional supplementation (Gupta et al., 2022). Phospholipid phytosomes significantly improve the solubility, bioavailability, and stability of plant extracts by leveraging the hydrophilic and lipophilic properties of phospholipids (Gnananath et al., 2017). Studies have shown that API-phospholipid phytosomes (APLC) significantly improved the water solubility, dissolution, oral bioavailability, and *in vivo* antioxidant activity of API compared to pure API (Telange et al., 2017). This suggests that phospholipid phytosomes are a potential strategy to improve the delivery of API and other similar poorly water-soluble plant components. Additionally, magnesium complexes as drug carriers can also enhance drug stability and bioavailability. Research has shown that API-magnesium complexes can reduce oxidative stress and inflammatory responses in H_2_O_2_-treated HSCs, suggesting their potential as a promising hepatoprotective agent (Pan et al., 2020).

In summary, these advanced drug delivery systems effectively overcome the limitations of API in terms of bioavailability, water solubility, and drug interactions, significantly enhancing its efficacy and safety in clinical applications. Future research should continue to focus on optimizing these technologies, exploring new carrier materials and delivery strategies to further enhance the clinical application potential of API. The compositions and achieved improvements of the new dosage form of API are presented in Table 8.

**TABLE 8:** Compositions and achieved improvements of the new dosage form of API.

References	Composition	Average particle size (nm)	Zeta potential (mV)	Drug loading efficiency (%)	Encapsulation efficiency (%)	Improvements
APINp3 (Bhattacharya et al., 2018)	API: Polymer = 1:3.3	270.00	−4.21	19.14 ± 0.16	82.96	Significantly increased API levels in blood and liver
API-SNPs (Zarei et al., 2021)	API: 3 g; AgNO_3_: 95 mL (1 mM)	93.94	34.80	N/A	N/A	Enhanced anticancer, antibacterial and antioxidant activities
API-NPs (Ganguly et al., 2021)	API: 12.5 mg; PLGA: 125 mg; Poloxamer 188: 250 mg	110.00 ± 25.00	−25.00 ± 1.30	5.10 ± 0.90	70.30 ± 2.60	Improved therapeutic efficacy achieved by active liver targeting
API-GAL-NPs (Ganguly et al., 2021)	API: 12.5 mg GAL-PLGA: 125 mg Poloxamer 188: 250 mg	129.00 ± 16.00	−14.00 ± 0.90	5.30 ± 0.60	75.40 ± 1.20	
API-CNP (Ding et al., 2014)	API: CNP = 1:6	N/A	N/A	N/A	N/A	Significantly improved the oral bioavailability
API-MSN (Huang et al., 2019a)	API: MSN = 1:6	49.00	−15.00	29.71	42.27	Improved the dissolution performance and the oral relative bioavailability
API-NPs (Lv, 2016)	API: PVP: TPGS = 2:1:1	83.39	−19	N/A	N/A	Significantly increased the bioavailability of API
NLCs (Dhara et al., 2023)	API: Cholesterol: Soya lecithin = 5:25:70	20.00	1.16	4.59 ± 0.02	91.86	Significantly improved target distribution of API
PEG-NLCs (Dhara et al., 2023)	API: Cholesterol: Soya lecithin: DSPE-PEG-2000 = 5:25:60:10	100.00	−55.9	4.38 ± 0.04	87.6	
Apt-NLCs (Dhara et al., 2023)	API: Cholesterol: Soya lecithin: DSPE-PEG-2000 = 5:25:60:10 (Functionalized with aptamer, AS1411)	155.00	−22.4	4.33 ± 0.05	86.6	
API-casein nanoparticless (Wang, 2022)	API: Casein = 3:5	238.70 ± 2.10	−7.48 ± 0.24	23.37 ± 2.51	48.56 ± 3.45	Significantly improved water solubility and bioavailability
API-magnesium complex (Pan et al., 2020)	API: Mg (AC)_2_ = 2:1	N/A	N/A	N/A	N/A	As a potential therapeutic drug in liver disease
APLC (Telange et al., 2017)	API: 1,4-dioxane-methanol = 14:6	107.08 ± 1.30	−22.35 ± 0.30	N/A	N/A	Significantly improved solubility, oral bioavailability and pharmacological properties of API
API-NPs (Yu, 2015)	API: PVP: TPGS = 2:1:1	83.39	−19	44.4 ± 0.06	N/A	Significantly increased the absorption of API
API-loaded polymeric micelles (Zhai et al., 2013)	API; P123; Solutol HS15	16.90	−5.87	1.32	96.36	Possessed a sustained release property
PLGA-encapsulated-nano-API (Das et al., 2013)	PLGA: API = 5:1	101.30 ± 0.00	−12.10 ± 0.00	N/A	87.25 ± 0.01	Reduced toxicity with higher efficacy and effectiveness
AP-LNC [192]	API: 20 mg; Lecithin: 30 mg; NaCl: 35 mg	46.10	−28.18	1.26	95.70	Significantly improved the dispersion or solubility of API in water
AP-SLN (Li, 2008)	API: Glycerol monostearate: Pluronic F68: Lecithin = 1:100:50:50	135.00 ± 18.00	−18.90	N/A	63.11 ± 0.64	Significantly improved bioavailability

Note: APINp, API loaded nanoparticles; API-SNPs, API, based synthesized silver nanoparticles; API-NPs, API nanoparticles; API-GAL-NPs, API galactose nanoparticles; CNP, carbon nanopowder; API-MSN, API, of solid dispersion of mesoporous silica nanoparticles; PVP, polyvinyl pyrrolidone; TPGS, d-alpha tocopheryl polyethylene glycol succinate; NLCs, API-encapsulated plain nanoliposomes; PEG-NLCs, PEGylated NLCs; Apt-NLCs, aptamer functionalized PEGylated NLCs; APLC, API-phospholipid phytosome; PLGA, poly (lactic-co-glycolide acid); AP-LNC, API-lipid nanocapsules; HP-β-CD, hydroxypropyl-β-cyclodextrin; AP-SLN, API solid lipid nanoparticles.

## 10 Discussion and future perspective

**FIGURE 5:**
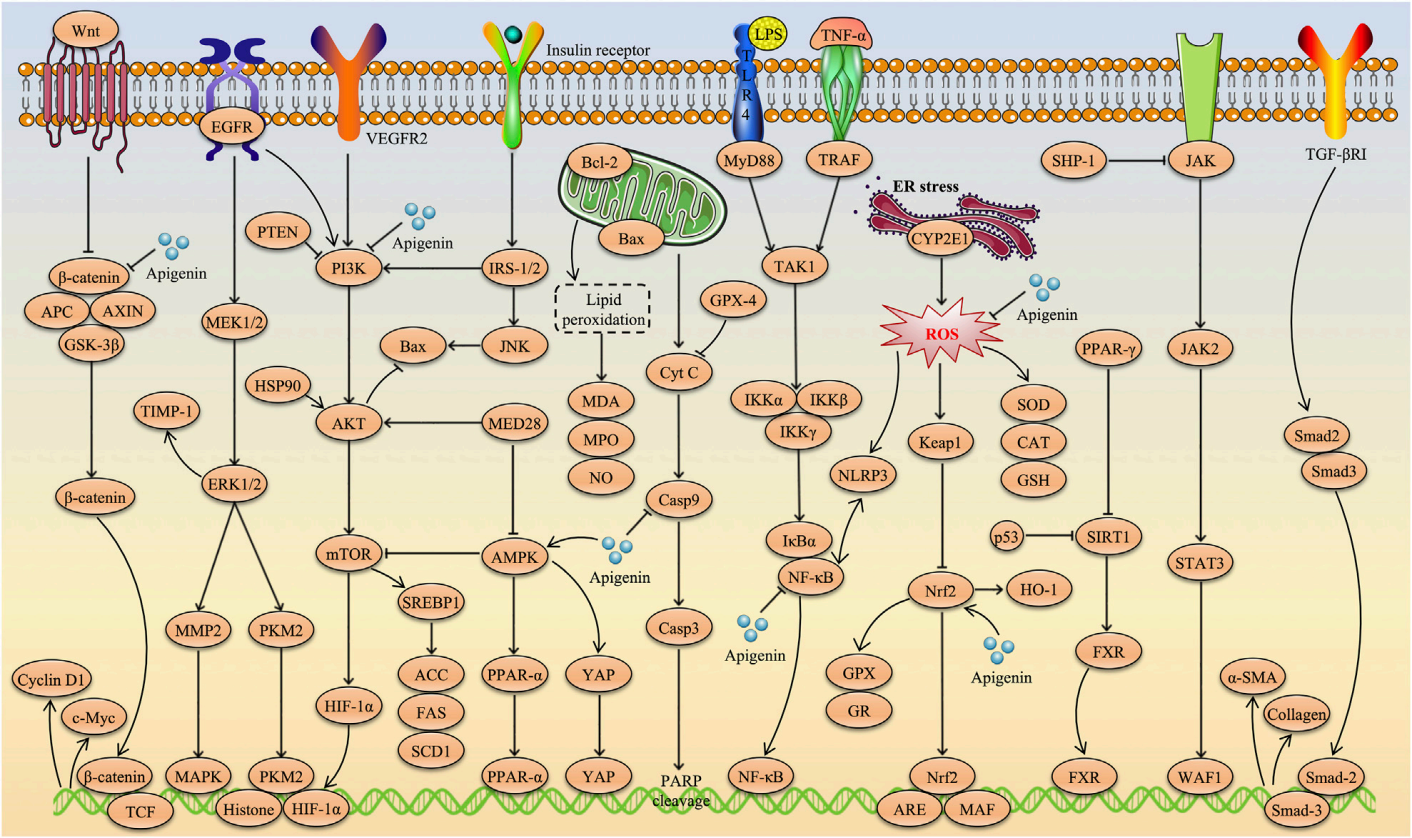
Molecular pathways of hepatoprotective effect of API.

As a naturally occurring flavonoid, API has garnered extensive academic interest in recent years due to its exceptional hepatoprotective properties. A substantial body of research evidence suggests that API holds significant potential in the prevention and treatment of various liver diseases, including liver injury caused by multiple factors, NAFLD/NASH, liver fibrosis, and HCC. The hepatoprotective effects of API are mediated through multiple molecular mechanisms, including the inhibition of inflammation, alleviation of hepatic oxidative stress, improvement of insulin resistance, promotion of fatty acid oxidation, reversal of macrophage polarization, regulation of bile acid metabolism, and the inhibition of HCC cell proliferation, differentiation, and induction of apoptosis. Importantly, signaling pathways such as Nrf2/HO-1, NF-κB, PI3K/Akt/mTOR, XO/NLRP3, Wnt/β-catenin, FXR, TGF-β1/Smad3, AMPK/SREBP, PPARα/γ, MAPKs, Caspases, and JAK2-STAT3 are considered critical targets through which API exerts its hepatoprotective effects. These findings provide robust scientific evidence for API as a potential therapeutic agent for liver diseases and lay a solid foundation for further exploration of its clinical application prospects. The molecular pathways of hepatoprotective effect of API are shown in Figure 5.

In terms of toxicity, existing experiments indicate that API exhibits low toxicity in both *in vitro* and *in vivo* studies. Even at relatively high doses, it does not induce significant adverse effects, demonstrating good safety. However, the pharmacokinetic properties of API remain challenging. Studies have shown that API has poor water solubility and low oral bioavailability, which may affect its absorption efficiency and ultimately its therapeutic efficacy *in vivo*. Furthermore, API’s slow metabolism and excretion may lead to accumulation in the body, potentially increasing toxicity risks or drug interactions. Although some studies have aimed at improving the pharmacokinetic properties of API, in-depth research on its absorption, distribution, metabolism, and excretion mechanisms remains limited, necessitating further studies to optimize its clinical application potential. To overcome these challenges, researchers are actively developing various drug delivery systems, such as nanoparticles, nanosuspensions, solid dispersions, and phospholipid phytosomes, to enhance the solubility and bioavailability of API, thereby increasing its therapeutic efficacy. Future studies should continue to explore and optimize these delivery systems to improve API’s drug characteristics and expand its clinical application prospects.

In light of these challenges, to effectively translate API into a clinically effective treatment for liver diseases, we propose the following recommendations:

(1) Further elucidation of mechanisms of action: Although the multiple biological activities of API have been confirmed, its specific mechanisms of action in liver diseases remain unclear. Future research should focus on elucidating its molecular mechanisms in oxidative stress, inflammatory responses, fibrosis progression, and the gut-liver axis to support its clinical application. Moreover, the integration of cutting-edge technologies such as single-cell sequencing, genomics, and transcriptomics in future studies could aid in revealing the comprehensive mechanisms of API, providing new perspectives for its application in the treatment of liver diseases.
(2) Toxicity evaluation and safety assessment: While API has demonstrated low toxicity in existing experiments, its long-term safety requires further evaluation. Future studies should systematically assess the safety of API in large-scale clinical trials and long-term toxicity studies, particularly at high doses and with prolonged use, to observe its potential impacts on the liver and other organs. In-depth toxicological research will ensure the safety of API in clinical applications.
(3) Drug delivery and bioavailability enhancement: The low bioavailability of API poses a significant obstacle to its clinical application. Future research should focus on developing various drug delivery systems, such as nanoparticles, nanosuspensions, and solid dispersions, to improve the solubility, bioavailability, and targeting capability of API. Additionally, exploring multifunctional carrier systems with combined functionalities, such as targeted delivery, controlled release, and imaging monitoring, could further enhance the therapeutic efficacy and clinical application prospects of API.
(4) Structural modification and prodrug design: Structural modifications of API or the design of prodrugs could enhance its pharmacological activity, stability, and absorption efficiency. For example, the addition of hydrophilic groups could improve its water solubility, thereby enhancing absorption efficiency; the introduction of specific chemical groups could enhance its anti-inflammatory or anti-fibrotic activity. Prodrug design could leverage enzymatic reactions within the body to release active API at the target site, thereby improving its efficacy.
(5) Clinical translation research and large-scale trials: Although current research has demonstrated the potential of API in hepatoprotection, large-scale clinical trials are lacking. Future studies should conduct large-scale, multicenter, randomized controlled clinical trials to validate the efficacy and safety of API in various liver diseases, such as NAFLD, liver fibrosis, and liver cancer. These clinical trials should cover diverse patient populations and include long-term follow-up to evaluate efficacy and safety, laying the groundwork for the clinical promotion of API.
(6) Exploration of combination therapy strategies: Future research should investigate combination therapy strategies involving API and other drugs, such as antiviral agents, antifibrotic drugs, or antioxidants, to evaluate their synergistic effects and potential drug interactions. Combination therapy may enhance overall efficacy, reduce the dosage and side effects of single drugs, and achieve better therapeutic outcomes.

In conclusion, this study comprehensively reviews the source, physicochemical properties, hepatoprotective effects, molecular mechanisms, toxicity, pharmacokinetics, and current development status of new formulations of API, while deeply exploring the challenges and opportunities in current research. Through this systematic summary, this study lays a solid theoretical foundation for further understanding the hepatoprotective effects and clinical application value of API, and it is expected to provide guidance for future research and application.

## Glossary

API: apigenin
APAP: acetaminophen
NAFLD: non-alcoholic fatty liver disease
ALD: alcoholic liver disease
HCC: hepatocellular carcinoma
Nrf2: nuclear factor erythroid 2-related factor 2
NF-κB: nuclear factor kappa-B
PI3K: phosphatidylinositide 3-kinase
Akt: protein kinase B
mTOR: mammalian target of rapamycin
NLRP3: NOD-like receptor family pyrin domain containing 3
FXR: farnesoid X receptor
TGF-β1: transforming growth factor-β1
Smad3: mothers against decapentaplegic homolog 3
PPARα: peroxisome proliferator-activated receptor alpha
APAP: acetaminophen
NAPQI: N-acetyl-p-benzoquinone imine
GR: glutathione reductase
GSH: glutathione
MTX: methotrexate
ALT: alanine aminotransferase
AST: aspartate aminotransferase
CAT: catalase
ALP: alkaline phosphatase
GSH-Px: glutathione peroxidase
SOD: superoxide dismutase
TNF-α: tumor necrosis factor α
IL-1β/6: interleukin-1β/6
MDA: malondialdehyde
CYC: cyclophosphamide;
ROS: reactive oxygen species
LPO: lipid peroxidation
MPO: myeloperoxidase
Bax: Bcl-2-associated X
Bcl-2: B-cell lymphoma-2
Fu: Furan
CCl4: carbon tetrachloride
DEHP: Di (2-ethylhexyl) phthalate
PbAc: lead acetate
NDEA: N-nitrosodiethylamine
LPS: lipopolysaccharide
D-GalN: D-Galactosamine
I/R: Ischemia-reperfusion
NiONPs: Nickel oxide nanoparticles
PA: palmitic acid
NASH: nonalcoholic steatohepatitis
OA: oleic acid
XO: xanthine oxidase
CTSB: cathepsin B
AMPK: AMP-activated protein kinase
FAS: fatty acid synthase
JAK2: Janus kinase 2
STAT3: signal transducer and activator of transcription 3
TC: total cholesterol
TG: triglyceride
ECM: extracellular matrix
HSCs: hepatic stellate cells
BDL: bile duct ligation
MMP2/8: matrix metalloproteinase 2/8
TIMP1: tissue inhibitors of metalloproteinase 1
iNOS: inducible nitric oxide synthase
NO: nitric oxide
ERK: extracellular signal-regulated kinase
H2O2: hydrogen peroxide
TLR4: Toll-like receptor4
DDC: 3,5-diethoxycarbonyl-1,4-dihydro-2,4,6-trimethylpyridine
MAPK: mitogen-activated protein kinases
IFN-I: type I interferon
miRNAs: microRNA
SHP-1: Src homology 2 domain-containing protein tyrosine phosphatase 1
EVs: extracellular vesicles
EMT: epithelial-mesenchymal transition
SOR: sorafenib
DOX: doxorubicin
PTX: paclitaxel
CDDP: cisplatin
5-FU: 5-Fluorouracil
CHR: chrysin
HPLC-MS/MS: high-performance liquid chromatography-tandem mass spectrometry
UPLC-MS/MS: ultra-performance liquid chromatography-tandem mass spectrometry
DEN: diethylnitrosamine
